# A comprehensive metabolite fingerprint of fibrostenosis in patients with Crohn’s disease

**DOI:** 10.1038/s41598-023-50461-1

**Published:** 2023-12-27

**Authors:** Simon Bos, Triana Lobatón, Martine De Vos, Sophie Van Welden, Vera Plekhova, Ellen De Paepe, Lynn Vanhaecke, Debby Laukens

**Affiliations:** 1 Department of Internal Medicine and Pediatrics, Ghent University, C. Heymanslaan 10, 0MRB2, 9000 Ghent, Belgium; 2 VIB Center for Inflammation Research, Ghent, Belgium; 3 Department of Gastroenterology, Ghent University Hospital, Ghent, Belgium; 4 Department of Translational Physiology, Infectiology and Public Health, Ghent University, Ghent, Belgium; 5 Institute for Global Food Security, Queen’s University, Belfast, Northern Ireland UK

**Keywords:** Crohn's disease, Molecular medicine, Predictive markers

## Abstract

Intestinal fibrostenosis in patients with Crohn’s disease (CD) is a common and untreatable comorbidity that is notoriously difficult to monitor. We aimed to find metabolites associated with the presence of fibrostenosis in patients with CD using targeted and untargeted metabolomics analyses of serum and primary cell cultures using hyphenated ultra-high performance liquid chromatography high-resolution mass spectrometry. Targeted metabolomics revealed 11 discriminating metabolites in serum, which were enriched within the arginine and proline metabolism pathway. Based on untargeted metabolomics and discriminant analysis, 166 components showed a high predictive value. In addition, human intestinal fibroblasts isolated from stenotic tissue were characterized by differential levels of medium-chain dicarboxylic acids, which are proposed as an energy source through beta-oxidation, when oxidative phosphorylation is insufficient. Another energy providing pathway in such situations is anaerobic glycolysis, a theory supported by increased expression of *hexokinase 2* and *solute carrier family 16 member 1* in stenotic fibroblasts. Of interest, four (unannotated) metabolic components showed a negative correlation with *hexokinase 2* gene expression. Together, this study provides a discriminative metabolic fingerprint in the serum and in intestinal fibroblasts of stenotic and non-stenotic patients with CD suggestive for increased production of building blocks for collagen synthesis and increased glycolysis.

## Introduction

Crohn’s disease (CD) is an idiopathic inflammatory bowel disease (IBD) defined by transmural inflammation of the intestinal tract, usually limited to the terminal ileum and/or the colon. Although the number of therapies controlling the inflammatory process in CD has increased tremendously over the past decades, loss of response to these drugs complicates therapeutic management. The typical disease course of CD is characterized by periods of symptoms alternating with periods of therapeutic control of disease symptoms, which inevitably leads to damage to the bowel and the accumulation of fibrotic tissue^1^. Consequently, more than 50% of patients with CD develop stricturing complications within the first ten years of diagnosis, ultimately leading to bowel obstruction^2^.

The standard treatment for bowel obstruction is surgery; however, recurrence rates are high. Despite advances in anti-inflammatory therapeutic strategies for CD, the incidence of fibrostenosis has not decreased, indicating that controlling inflammation alone is not sufficient to prevent fibrosis. Together with the lack of drugs to prevent the accumulation of fibrotic tissue in CD patients, the management of intestinal tissue damage is a major clinical challenge. Because fibrostenosis coincides with inflammatory tissue swelling, it is extremely difficult to adequately detect and/or quantify fibrosis in the gut using traditional techniques such as imaging or endoscopy^3^. In addition, the search for biomarkers of intestinal fibrosis has been unsuccessful, which was also concluded in a recent meta-analysis^4^. Not surprisingly, clinical trials for antifibrotic drugs are severely hampered by the lack of clinical or surrogate endpoints, further highlighting the need for biomarkers of intestinal fibrosis.

Metabolomics analysis represents an attractive and unexplored strategy for the discovery of circulating markers of fibrosis in serum or plasma. A key feature of fibrosis is the transition and accumulation of activated fibroblasts in the intestinal mucosa, which produce the collagens found in fibrotic tissue. During this activated cell state, the metabolic machinery of the fibroblast requires increased nutrients and energy^5^. Although knowledge of fibroblast phenotypes in the fibrotic gut is limited, we can draw parallels from other fibrotic diseases in which modulation of fatty acid metabolism and increased glutaminolysis have been shown to be critical for maintaining adequate collagen production in fibroblasts^6^. Several papers have reported that the Warburg effect, a well-known process in cancer biology that switches the energy production from oxidative phosphorylation to glycolysis despite the presence of oxygen, also occurs in fibroblasts found in fibrotic tissue^7–9^. Since many of the metabolites implicated in these processes are secreted from the fibroblasts, it can be expected that the local changes in cellular metabolism will also be reflected in the blood.

The state-of-the-art technique for performing metabolomics is (liquid) chromatography hyphenated to high-resolution mass spectrometry (HRMS). Due to its high resolving power and mass accuracy, HRMS can detect a wide range of low abundant metabolites, ensuring high sensitivity^10,11^. This mass spectrometric approach has been shown to be relevant for the detection of metabolic changes between IBD and healthy controls. For example, differences in branched chain amino acids have been reported in patients with CD and ulcerative colitis. Other divergent metabolites discovered by metabolomics in IBD include 3-hydroxybutyrate, glutamine, histidine, tryptophan, secondary bile acids, arachidonic acid, and leukotrienes^12^. While untargeted metabolomics analysis can generate (biomarker) fingerprints, such approach does not allow exact identification of all signals observed. Targeted approaches, during which typically in-house and functionally diverse metabolites are analysed together with the unknown samples, represent a valuable addition to the untargeted approach.

In this study, we applied targeted and untargeted UHPLC-HRMS metabolomics analyses to discover discriminating markers in the serum of CD patients with stenosis or uncomplicated disease at the time of sampling. In addition, untargeted metabolomics was used to screen for metabolite changes in patient-derived intestinal fibroblasts collected from stenotic versus non-stenotic regions, and to screen for overlap with the serum metabolic profile.

## Results

### Method validation for serum samples proves the method is fit-for-purpose

This research utilized a serum methodology derived from our previously developed and extensively validated in-house UHPLC-HRMS method for plasma analysis^13^. Since this method had already undergone comprehensive validation for plasma, the validation for serum specifically focused on assessing instrumental precision, as well as intra-assay and interday precision. For the targeted analysis, 144 standard reference metabolites were evaluated (Supplementary Table 1), of which 104 were accurately (mass deviation < 5 ppm) detected in serum, representing diverse chemical classes. For the targeted validation, instrumental precision was considered good as 76.92% (80/104) of the metabolites showed a coefficient of variance (CV) ≤ 20%. Similarly, 74.04% (77/104) had a CV ≤ 20% for intra-assay precision. For inter-day precision, this percentage was 68.27% (71/104). For the untargeted validation, a total of 4164 components were identified with an intensity > 500,000 a.u., of which 2616 in positive mode and 1548 in negative mode. In positive ionization, respectively 92.47% (n = 2419), 89.11% (n = 2331) and 84.37% (n = 2207) of the components displayed a CV value ≤ 30% for the instrumental, intra-assay and inter-day precision. In negative ionization, CV values ≤ 30% were achieved for 92.70% (n = 1435), 92.05% (n = 1425) and 79.26% (n = 1227) components for instrumental, intra-day and inter-day precision, respectively. Overall, these analyses demonstrated accurate performance and acceptable metabolite coverage, ensuring that the extraction and analysis method is fit-for-purpose for both targeted and untargeted serum metabolomics.

### Targeted serum metabolomics reveals modulation of the arginine and proline metabolism in fibrostenotic Crohn’s disease

**Figure 1:**
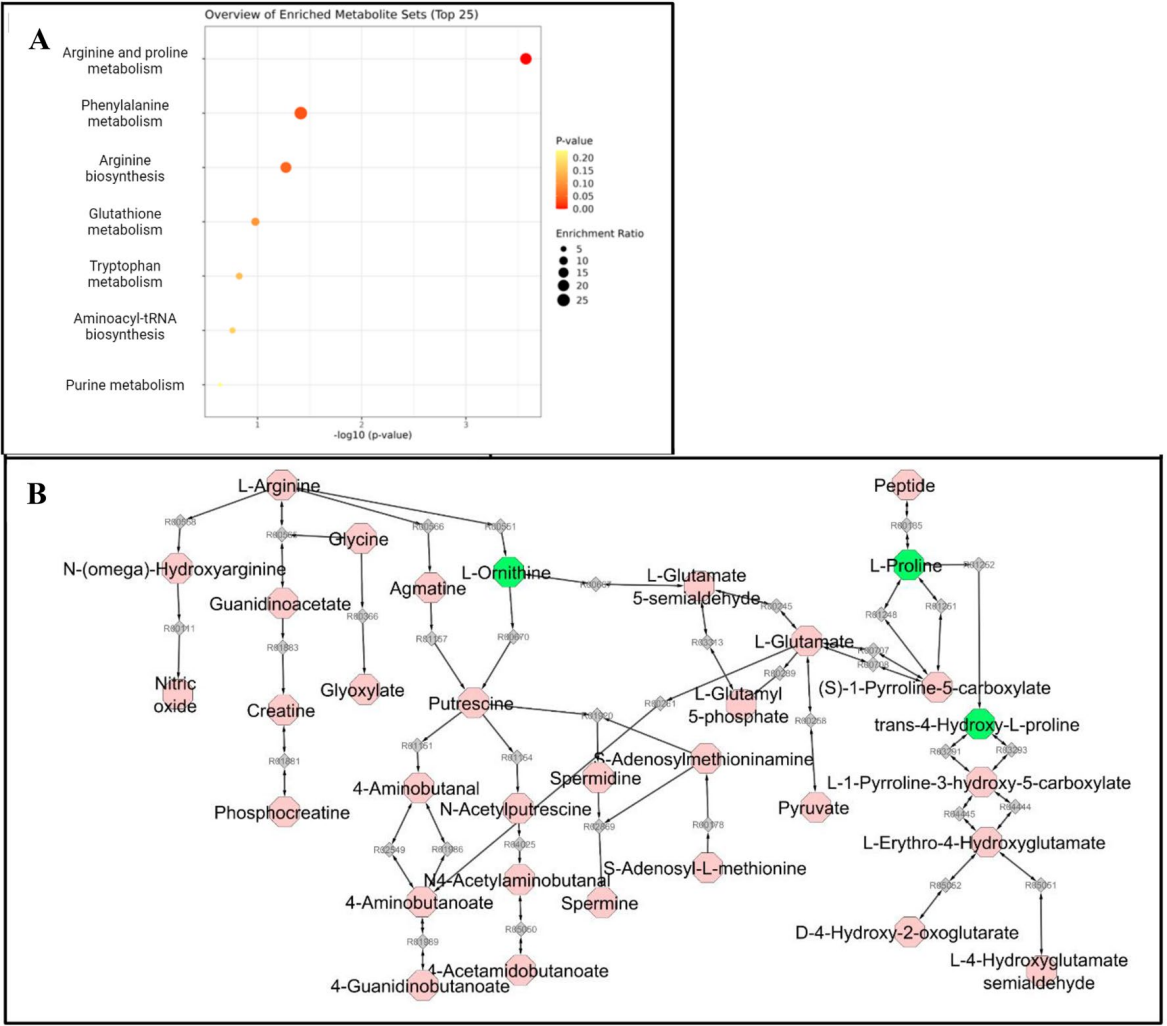
Enrichment analysis of the differential metabolites in the sera of stenotic (n = 28) *vs.* non-stenotic (n = 38) Crohn’s Disease (CD) patients based on targeted analysis. (**A**) Overview of the pathways enriched in 11 differential metabolites, colored by p-value and sized by enrichment ratio. (**B**) The arginine and proline metabolic pathways generated three differential metabolites (green), i.e., l-ornithine, l-proline, and trans-4-hydroxy-l-proline (created with MetScape in Cytoscape).

A total of 66 patients were included in the study, 28 in the stenotic group and 38 in the non-stenotic group (Supplementary Table 2). Groups were matched for age at CD diagnosis, gender, anti-TNF use, Montreal classification and symptomatology (defined by the presence of inflammation on endoscopy, CT or MRI and/or CRP > 10 mg/L). Targeted metabolomics analysis on 104 analytes yielded 11 differential metabolites (Table 1) with a p-value < 0.05, of which one signal represented the combined abundance of deoxycholic acid (DCA), and/or chenodeoxycholic acid (CDCA) as they have similar *m/z*-values and retention times. All 11 metabolites were increased in the stenotic group compared to the non-stenotic group. Pathway enrichment analysis based on the KEGG human pathway database, revealed that the arginine and proline metabolism was significantly affected (Fig. 1A, p = 2.58e^−4^, FDR = 0.022) as three of the 11 targeted metabolites (l-proline, l-ornithine, and trans-4-hydroxy-l-proline) occurred in this pathway, with a total pathway impact of 0.249 (Fig. 1B).

**Table 1:** Targeted serum metabolomics analysis of patients with stenotic (n = 28) *vs.* without stenotic (n = 38) regions.

Metabolite	*m/z* (Da)	RT	Ionisation	Group	p-value
l-Proline	116.07061	1.0	[M+H]^+^	Stenosis	6.00e^−4^
trans-4-Hydroxy-l-proline	132.06552	0.9	[M+H]^+^	Stenosis	1.20e^−2^
Deoxycholic acid or chenodeoxycholic acid	391.28534	13.0	[M−H]^−^	Stenosis	1.50e^−2^
2-Phenylethylamine	122.09643	6.6	[M+H]^+^	Stenosis	2.00e^−2^
2-Piperidinone	100.07569	5.0	[M+H]^+^	Stenosis	2.20e^−2^
l-Ornithine	133.09715	0.8	[M+H]^+^	Stenosis	2.40e^−2^
Allantoin	159.05127	1.0	[M+H]^+^	Stenosis	3.40e^−2^
3-Indoleacetic acid	176.07061	10.8	[M+H]^+^	Stenosis	3.80e^−2^
Glycolitocholic acid	434.32649	13.0	[M−H]^−^	Stenosis	3.80e^−2^
Lysophosphatidylcholine C18:1	522.35542	13.7	[M+H]^+^	Stenosis	4.30e^−2^
Hexanoic acid	117.09101	11.8	[M+H]^+^	Stenosis	4.90e^−2^

RT, retention time. Wilcoxon rank sum test was applied, with continuity correction when required.

### Serum metabolic fingerprint of Crohn’s disease patients discriminate between patients with and without fibrostenosis

Untargeted metabolomics of the serum samples yielded a total of 5,959 components. Notably, the subsequent quality control (QC) samples exhibited clustering in the unsupervised principal component analysis (PCA) plot, therefore providing robust confirmation of the instrument’s precision (Supplementary Fig. 1).

**Figure 2:**
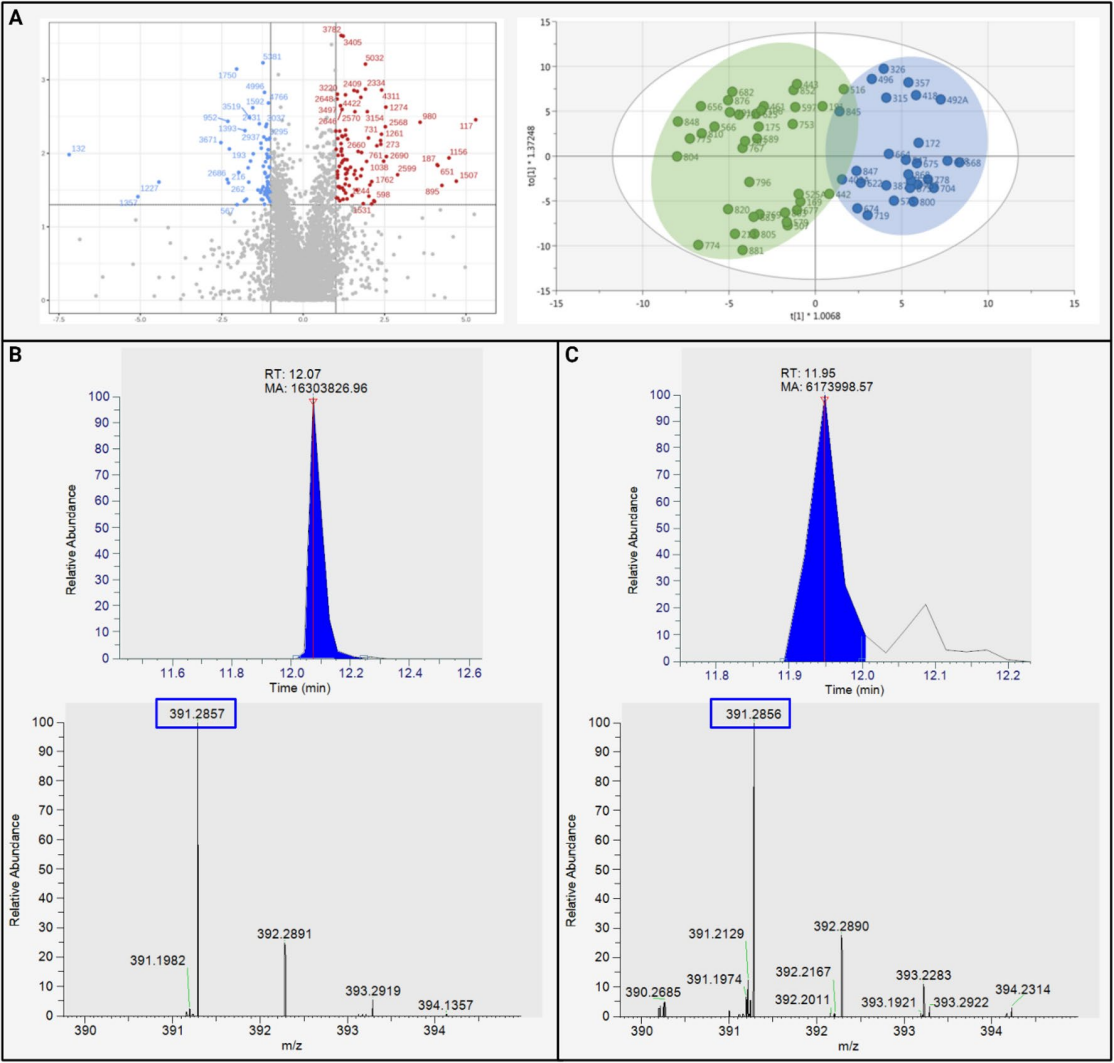
Untargeted metabolomics analysis of serum of stenotic (n = 28) and non-stenotic (n = 38) Crohn’s Disease (CD) patients. (**A**) Volcano plot of 5959 components, showing a total of 166 components that where up or down regulated (red and blue dots, respectively) with a p-value < 0.05 and an absolute fold change of more than |2| (left), and the score plot of the OPLS-DA model built with the 166 components retained from the volcano plot (right). (**B**) Chromatogram and MS/MS spectrum of ursodeoxycholic acid (UDCA) as analytical standard. (**C**) Chromatogram and MS/MS mass spectrum of UDCA in a serum sample. FC, fold change, OPLS-DA, orthogonal partial least squares discriminant analysis.

Of the 5959 detected components, 166 met the predetermined criteria (fold change >|2|, p-value < 0.05) as they were differentially abundant between the stenotic *vs.* non-stenotic patient samples (Fig. 2A, Supplementary Table 3). Ninety-two components increased in the serum of stenotic patients, whereas 74 decreased. Using these 166 components, an OPLS-DA model was built that showed high predictive accuracy and could be validated (Fig. 2A), with the following model parameters: R^2^(Y) = 0.78, Q^2^(Y) = 0.55, CV-ANOVA p-value 1.48e^−8^ and a valid permutation test (n = 100). Eleven components with a variable importance in projection > 1, a Jack-knifed confidence interval not across 0, and an S-plot covariance >|0.05| were considered most discriminative, of which one was identified at MSI1 according to Schymanski et al.^14^, by matching with an authentical chemical standard present in our reference in-house database, i.e., ursodeoxycholic acid (UDCA) (Table 2, Fig. 2 and Supplementary Fig. 3). Although both patient groups were matched for symptomatology (Supplementary Table 2), we questioned if untargeted metabolomics could distinguish between the patients’ inflammatory status, i.e., remission (n = 26) *vs.* active disease (n = 40), by unsupervised modelling with PCA. As no discernable clustering was observed (Supplementary Fig. 2), no matching based on inflammatory state was considered for subsequent data analysis.

**Table 2:** Untargeted metabolomics analysis of serum samples of patients with stenotic (n = 28) *vs.* non-stenotic (n = 38) Crohn's Disease .

Comp ID	*m/z* (Da)	RT (min)	Ionisation	Fold Change	Group	p-value	Metabolite name
937	242.21135	14.8	[M−H]^−^	− 2.10	No stenosis	8.92e^−3^	ND
1274	471.24197	11.6	[M−H]^−^	+ 0.17	Stenosis	2.40e^−3^	SO_3_ adduct of UDCA
3037	512.40344	15.5	[M−H]^−^	− 2.17	No stenosis	4.00e^−3^	^13^C isotope ID 3561
3561	511.40009	15.3	[M−H]^−^	− 2.12	No stenosis	2.08e^−2^	ND
4072	465.35846	15.1	[M−H]^−^	+ 2.25	No stenosis	1.88e^−2^	Component 4073
4073	465.35840	14.9	[M−H]^−^	+ 2.03	No stenosis	6.20e^−3^	Component 4072
4311	391.28571	12.0	[M−H]^−^	+ 0.19	Stenosis	1.38e^−3^	UDCA^a^
4766	391.28540	15.1	[M−H]^−^	− 2.09	No stenosis	2.00e^−3^	ND
4841	409.29590	14.8	[M−H]^−^	− 2.26	No stenosis	1.82e^−2^	ND
4946	678.40460	11.8	[M−H]^−^	− 2.89	No stenosis	1.02e^−2^	ND
4996	441.35867	14.9	[M−H]^−^	− 2.28	No stenosis	1.50e^−3^	ND

Comp ID: component identification number; RT: retention time. T-test was applied on log-transformed and pareto-scaled data. ^a^Matched with authentical chemical standard (MSI1); UDCA, ursodeoxycholic acid; + increased in stenotic group.

### Metabolic fingerprinting of stenotic intestinal fibroblasts identifies differential expression of medium-chain dicarboxylic acids

To provide more mechanistic insights, untargeted metabolomics was performed on human intestinal fibroblasts. For this purpose, primary intestinal fibroblasts were isolated from stenotic and non-stenotic regions of the same resection specimen (n = 7 donors; Supplementary Table 4) and cultured until reaching confluence. A total of respectively 830 and 463 components were extracted from supernatant and the corresponding lysate, which were all retained for further statistical analysis. Unsupervised PCA modelling showed acceptable clustering of subsequent QC samples, thereby confirming good instrumental precision (Supplementary Figs. 4 and 5). Considering the small number of components retained, only univariate statistical analysis was performed. The latter revealed eight components significantly discriminating the cell lysate of stenotic *vs.* non-stenotic regions (Table 3 and Supplementary Fig. 6). Similar analysis of the supernatant data revealed 19 discriminating components between stenotic and non-stenotic fibroblasts (Table 4 and Supplementary Fig. 7a and b). The *m/z* and retention time of these components were compared to our in-house standard database, and, upon match, the mass spectrometric data was benchmarked against the reference standard data. This approach resulted in two components with identification confidence MSI1, namely suberic acid in cell lysate and adipic acid in supernatants (Supplementary Figs. 8 and 9). Both compounds are medium-chain, even numbered dicarboxylic acids, that can serve as energy supply, representing an alternative source of succinate in gluconeogenesis/glucose metabolism^15^. An additional seven components in supernatant received putative identifications, based on ChemSpider and m/zCloud database searches by Compound Discoverer (Table 4).

**Table 3:** Untargeted metabolomics analysis of stenotic versus non-stenotic fibroblast cell lysates.

Comp ID	*m/z* (Da)	RT (min)	Ionization	t.stat	Group	p-value	Metabolite ID	*P*corr. *HK2*	*P*corr. *SLC16A1*
103	141.95859	0.72	[M+H]^+^	− 2.18	Stenosis	4.96e^−2^	ND	− 0.060	− 0.016
139	384.93527	0.86	[M−H]^−^	− 2.90	Stenosis	1.33e^−2^	ND	0.25	0.40
158	150.88542	1.10	[M−H]^−^	− 2.77	Stenosis	1.70e^−2^	ND	0.12	0.34
164	452.92252	0.89	[M−H]^−^	− 2.75	Stenosis	1.76e^−2^	ND	0.24	0.40
201	142.94810	0.90	[M+H]^+^	− 2.71	Stenosis	1.88e^−2^	ND	0.11	0.42
289	164.94647	1.42	[M−H]^−^	− 2.22	Stenosis	4.61e^−2^	ND	− 0.084	0.065
517	175.09645	10.04	[M+H]^+^	− 2.58	Stenosis	2.43e^−2^	Suberic acid^a^	0.19	− 0.24
754	197.88908	0.71	[M−H]^−^	− 2.70	Stenosis	1.94e^−2^	ND	0.21	0.057

Comp ID: component identification number; RT: retention time. ^a^Matched with authentical chemical standard (MSI1). (−) tstat: increased in stenotic group.

**Table 4:** Untargeted metabolomics analysis of stenotic versus non-stenotic fibroblast supernatant.

Comp ID	*m/z* (Da)	RT (min)	Ionizat-ion	t.stat	Group	p-value	Metabolite ID	*P*corr. *HK2*	*P*corr. *SLC16A1*
2	239.1058	1.28	[M+H]^+^	+ 3.52	No stenosis	4.25e^−3^	ND	− 0.032	− 0.47
56	159.06509	8.56	[M−H]^−^	+ 2.37	No stenosis	3.50e^−2^	ND	− 0.59**	0.36
98	145.04933	7.00	[M−H]^−^	+ 2.66	No stenosis	2.09e^−2^	Adipic acid^a^	− 0.31	0.39
113	137.02307	7.86	[M−H]^−^	+ 3.09	No stenosis	9.29e^−3^	3/4-Hydroxy-benzoic acid^b^	− 0.56*	0.11
152	113.96394	0.65	[M+H]^+^	+ 2.27	No stenosis	4.23e^−2^	ND	− 0.17	0.082
309	171.06519	8.09	[M+H]^+^	+ 2.28	No stenosis	4.18e^−2^	3,4-Dihydroxy-phenylglycol^b^	− 0.036	− 0.21
370	111.04433	7.01	[M+H]^+^	+ 3.23	No stenosis	7.24e^−3^	Hydroquinone^b^	− 0.31	0.21
427	129.05467	7.00	[M+H]^+^	+ 2.74	No stenosis	1.79e^−2^	2,5-bis-(Hydroxy-methyl)furan^b^	− 0.62**	0.34
461	115.0392	3.96	[M+H]^+^	+ 2.83	No stenosis	1.51e^−2^	cis-Acetyl-acrylic acid^b^	− 0.55*	0.075
476	173.02094	1.08	[M+H]^+^	+ 4.40	No stenosis	8.70e^−4^	Glycerol-3-phosphate^b^	− 0.52*	− 0.30
510	145.04953	0.61	[M+H]^+^	+ 2.51	No stenosis	2.75e^−2^	Dimethyl fumarate^b^	0.010	− 0.46
533	204.12297	7.98	[M+H]^+^	+ 2.39	No stenosis	3.38e^−2^	ND	− 0.24	− 0.41
541	152.0706	3.57	[M+H]^+^	+ 2.43	No stenosis	3.20e^−2^	ND	0.14	− 0.034
558	207.91809	0.63	[M−H]^−^	− 2.31	Stenosis	3.95e^−2^	ND	− 0.022	− 0.020
709	143.03371	4.19	[M−H]^−^	+ 2.48	No stenosis	2.87e^−2^	ND	− 0.38	− 0.53*
739	165.01825	8.48	[M−H]^−^	+ 2.25	No stenosis	4.41e^−2^	ND	0.056	0.18
789	276.18021	11.01	[M+H]^+^	+ 2.44	No stenosis	3.11e^−2^	ND	− 0.19	0.18
809	204.12302	8.56	[M+H]^+^	+ 2.35	No stenosis	3.70e^−2^	ND	− 0.58**	− 0.26
818	556.44092	14.91	[M+H]^+^	+ 2.34	No stenosis	3.70e^−2^	ND	− 0.82**	0.17

Comp ID: component identification number; RT, retention time. ^a^Matched with authentical chemical standard (MSI1); ^b^matched with Compound Discoverer (MSI4). **p-value < 0.05; *p-value < 0.10. (+) tstat: increased in non-stenotic group; (−) tstat: increased in stenotic group.

### Metabolic fingerprinting of serum, fibroblast supernatant and cell lysate reveals nearly 30% overlap

**Figure 3:**
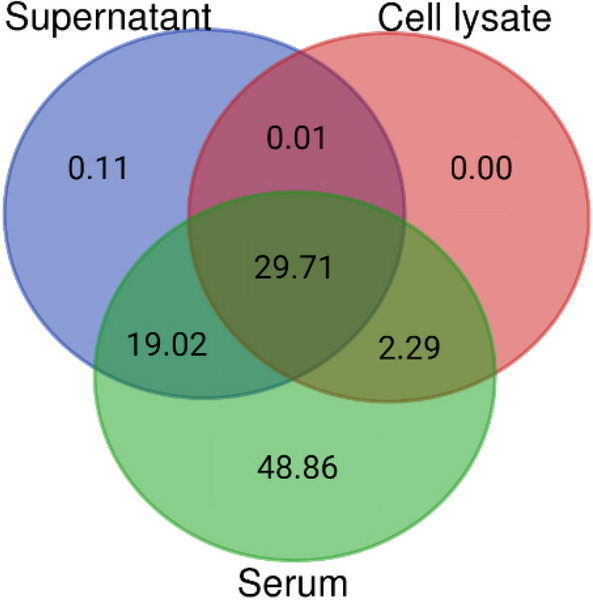
Venn diagram displaying the percentages of unique and overlapping metabolic components between serum, cell lysate and supernatant based on untargeted analysis.

In general, serum emerged as the biofluid containing the highest number of metabolic components under examination, comprising 99.88% of all detected components. Among these, 48.73% were shared with supernatants of cultured stenotic fibroblasts, and 32% were present in their cell lysates as well. Specifically, 48.86%, 0.11% and 0.00% of components were exclusive to serum, supernatant and cell lysate, respectively, whereas 29.71% of metabolites were observed in all three matrices (Fig. 3).

### Stenotic intestinal fibroblasts exhibit characteristics of increased glycolysis

**Figure 4:**
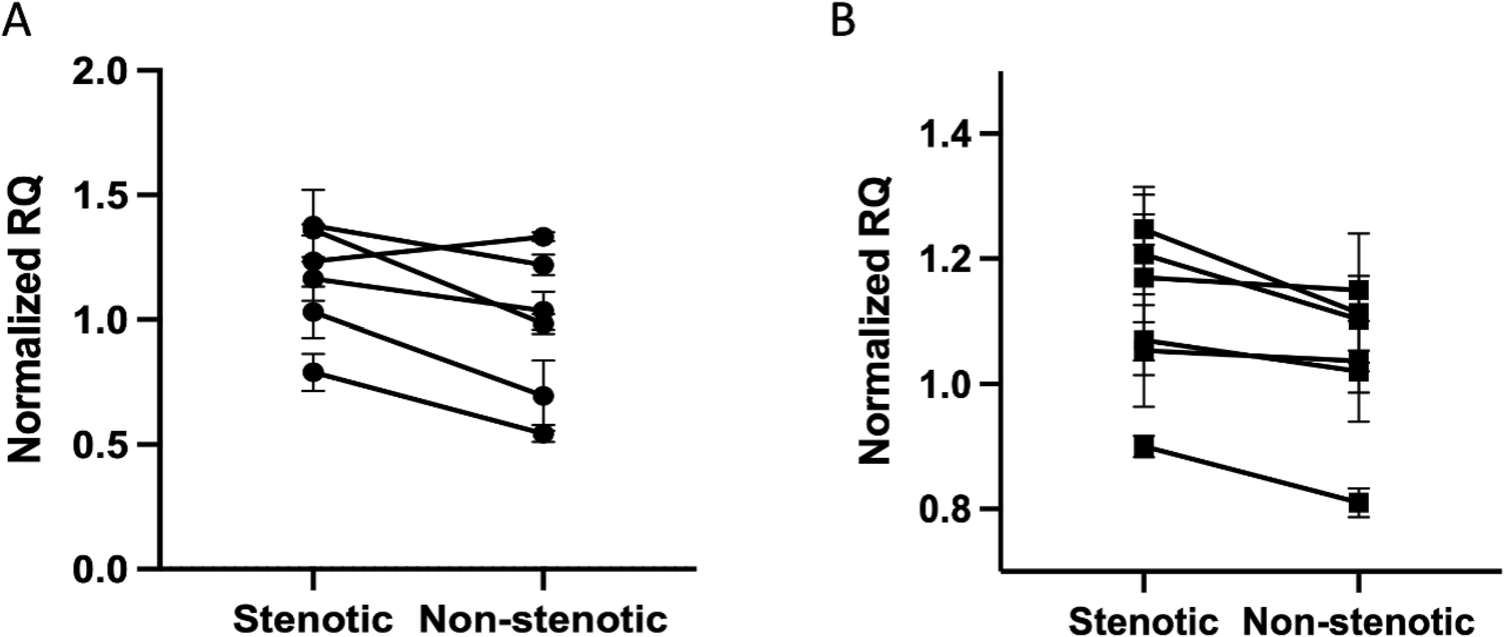
Increased glycolysis in intestinal fibroblasts isolated from the stenotic intestine. (**A**) expression of *hexokinase 2* and (**B**) t*he solute carrier family 16 member 1 *in fibroblasts isolated from the stenotic and non-stenotic area of the same donor patients. A paired t-test was performed; error bars represent the standard error of the mean. RQ, relative quantification.

As our untargeted analysis of fibroblast supernatant and cell lysate identified multiple metabolites related to the energy metabolism, and considering that switches in energy production from oxidative phosphorylation to glycolysis have been documented in fibroblasts within fibrotic tissue, despite the presence of oxygen^7–9^, we further sought to validate the hypothesized glycolytic switch in intestinal fibroblasts collected from a stenosis. The latter was evaluated by quantifying the transcript levels of the key enzyme regulating the first step of glycolysis, *hexokinase 2* (*HK2*) and of the transporter regulating the efflux of excess lactate, *solute carrier family 16 member 1* (*SLC16A1*). Both *HK2* and *SLC16A1* showed significantly higher expression in stenotic fibroblasts compared to the non-stenotic fibroblasts from the same donor (p = 0.042 and p = 0.016, respectively, Fig. 4A,B).

**Figure 5:**
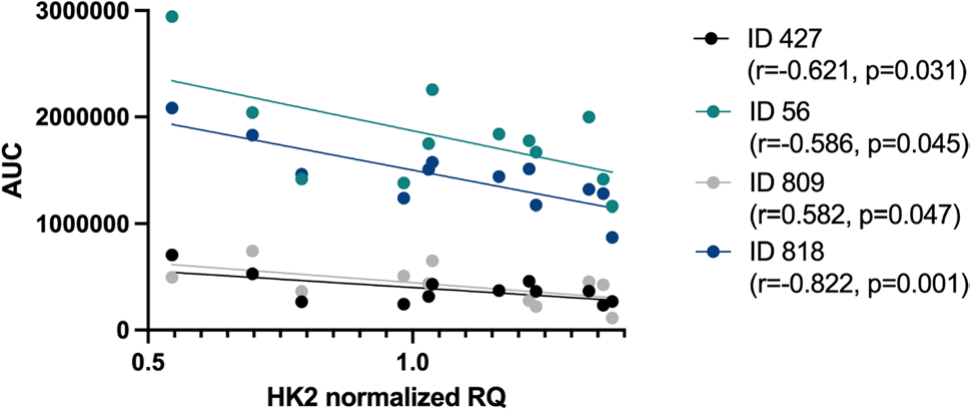
Differential components (ID 427, ID 56, ID 809, and ID 818) found in the supernatant of intestinal fibroblasts, show a significant negative correlation with hexokinase 2 (*HK2*) gene expression. Pearson correlation test performed with the resulting Pearson correlation coefficient. ID, identification; AUC, area under the curve; RQ, relative quantification.

We next aimed to correlate the expression of *HK2* and *SLC16A1* with components found in the supernatant and lysate of these cells. No significant correlations were observed for *SLC16A1*, nor for the cell lysates, whereas for supernatant, 4 components, i.e. ID 427 (r = − 0.621, p = 0.031), ID 56 (*r* = − 0.586, p = 0.045), ID 809 (r = − 0.582, p = 0.047) and ID 818 (r = − 0.822, p = 0.001) showed a significant negative correlation with *HK2* transcript levels (Tables 3 and 4, Fig. 5).

## Discussion

Metabolomics analysis of serum provided a fingerprint with 11 and 166 components (of which 11 with high discriminating value), respectively, that effectively discriminated CD patients with concomitant stenosis from those without stenosis. Most striking for the targeted metabolomics analysis of serum was the differential expression in the arginine and proline metabolism between patients with and without stenosis. In addition, untargeted metabolomics data of fibroblasts, including analysis of cell lysate and supernatant, isolated from the stenotic and non-stenotic regions of the intestine of patients with CD exhibited signs of an altered energy metabolism.

The pathway-enriched discriminating metabolites identified through targeted analysis of serum included increased l-proline, trans-4-hydroxy-l-proline and l-ornithine abundances in patients with stenotic CD. l-proline is a typical building block of collagen, and its abundance may reflect the intensity of collagen biosynthesis. In this regard, l-ornithine, combined with l-glutamate, is a precursor for the de novo synthesis of l-proline^16^. In addition, TGF-β, which is a potent fibroblast activator, induces mitochondrial redox stress by upregulating glucose and glutamine consuming energy metabolism to meet the bioenergetic cost of matrix protein production. In response to this increased energy metabolism, fibroblasts attempt to vent intermediate metabolites to other pathways before they can form damaging reactive oxygen species once anaerobic glycolysis takes over. Indeed, it has been shown that l-proline synthesis is an efficient way to vent these potentially harmful intermediates while also supporting the production of matrix proteins, another process induced by TGF-β^17^. In addition, the increased levels of trans-4-hydroxy-l-proline may indicate that remodeling of the extracellular matrix is ongoing, given that this metabolite is almost exclusively found in collagen, as it is formed upon hydroxylation of the collagen proline moiety. Of interest, trans-4-hydroxy-l-proline has been reported as a biomarker for liver and lung fibrosis^18,19^.

Untargeted serum metabolomics revealed an interesting fingerprint for stenosis as well. Next to a MIS1 level identification for UDCA, we can infer from the late retention times, high molecular masses, and negative ionization of the discriminative components that these are likely large, non-polar, lipid or apolipoprotein-like components. These findings would be consistent with the identification of lysophosphatidylcholine C18:1 from the targeted approach, and the recent recognition that lipid metabolism plays a critical role in the development of fibrotic tissue, as demonstrated by occurrence of creeping fat surrounding stenotic lesions in the gut^6,20,21^. These data also indicate that the search for new biomarkers may benefit from a more lipid-oriented approach, such as lipidomics.

We had anticipated to find overlapping discriminating metabolites in serum and the cell lysates and supernatant of intestinal fibroblasts from patients with stenotic disease, which would have added to the functional understanding of the identified fingerprints. Although we could not observe such overlap, the metabolomics analysis of the fibroblasts suggested a role for altered energy supply in the stenotic fibroblast compared to its non-stenotic counterpart. In the cell lysates of these fibroblasts, one of the distinguishing components was with high prediction (MSI1) identified as suberic acid, which was present in higher concentrations in the stenotic regions. This compound is a degradation product of oleic acid and has recently been shown to be involved in the increased collagen production in dermal fibroblasts after activation of the cAMP-Akt pathway^22^. In cell supernatant of stenotic regions, lower levels of adipic acid were identified with MSI1 confidence. Indeed, adipic acid is known to be involved in the biodegradation degree of collagen^23^. In addition, suberic acid and adipic acid are both dicarboxylic acids, that are also present in the urine of patients with fatty acid oxidation disorders^24,25^. Indeed, both dicarboxylic acids can serve as a source of succinate, implicating their participation in cellular energy metabolism^15^. In addition, a glycolytic switch was also confirmed by an increased gene transcription of *HK2*, a key enzyme involved in glycolysis, in the stenotic compared to the non-stenotic fibroblasts. In addition, we identified four discriminatory components that correlated with *HK2* transcription, suggesting an increased uptake of these metabolites associated with an increase in the glycolytic pathway.

Unfortunately, today’s molecular network suspect libraries can only annotate up to 20% of the fragmentation data of metabolites, which hampers the identification of metabolites^26^. Thus, further identification of the unidentified components will provide more mechanistic insight in intestinal fibrostenotic disease. While waiting for the number of annotated components to increase, the presented metabolic fingerprints for fibrostenosis in patients with CD is a good starting point for biomarker validation studies.

Overall, this study provided new insights into the metabolic changes occurring in CD patients with fibrostenosis, suggesting the facilitation of the production of building blocks for extracellular matrix expansion and a shift in energy production pathways in the fibroblasts found in the stenotic intestine. Once validated, the metabolite markers highlighted in this study could play a diagnostic role in identifying intestinal fibrosis in CD patients. This advancement would significantly aid in tailoring individualized treatment strategies. Furthermore, these biomarkers hold promise for the development and implementation of anti-fibrotic trials, marking a substantial step forward in the management of CD.

## Materials and methods

### Ethics

All studies were approved by the Ethics Committee of Ghent University Hospital (EC/2018/1123 with Belgian registration number B670201837383 and EC/2018/0952 with Belgian registration number B670201837001), and written informed consent was obtained from all participants. Experiments were performed in accordance with relevant guidelines and regulations.

### Patient selection

Serum samples were selected from a biobank of CD patients (Belgian reference number BB190100) with ileal or ileocolonic disease (Montreal L1 or L3). Samples were included in the ‘stenotic’ group based on luminal narrowing and pre-stenotic dilatation on MRI/CT at the time of sampling (n = 28). Patients in the ‘non-stenotic’ group were included if they were within 5 years of diagnosis and had no evidence of luminal narrowing on MRI/CT (n = 38). Active symptomatology was assessed by the presence of inflammation on endoscopy, CT or MRI and/or CRP > 10 mg/L. Exclusion criteria were liver disease or complications, infectious disease, human immunodeficiency virus infection, colorectal cancer, hormonal disorders and pregnancy. The selection of patients based on the criteria was agreed upon by two independent gastroenterologists (MDV and TL).

### Isolation and culture of fibroblasts from resection specimens

Primary human intestinal fibroblasts were isolated as described^27^. Briefly, mucosal samples were dissected from the stenotic and non-stenotic regions of freshly resected bowel segments from 7 donors (Supplementary Table 4) and washed in RPMI 1640 medium supplemented with 10% FBS, 10,000 U/mL penicillin, 10,000 µg/mL streptomycin, 25 µg/mL Fungizone, and 200 µg/mL gentamycin (Thermo Fisher, Waltham, MA, USA). Mucus and epithelial cells were removed by incubation of samples with DTT and EDTA for 30 min at room temperature and 37 °C, respectively, followed by enzymatic digestion with 25,000 U of collagenase type 2 (Worthington Biochemical, Lakewood, NJ, USA) and 30 µg/mL Dnase I (Roche, Mannheim, Germany) for 1 h at 37 °C. Cells were filtered sequentially through 100-µm and 70-µm filters (Greiner Bio-One, Vilvoorde, Belgium) and cultured in fibronectin-coated flasks in medium 106 supplemented with low-serum growth supplement (Thermo Fisher, Waltham, MA, USA), 10,000 U/mL penicillin, 10 µg/mL streptomycin and 50 µg/mL gentamycin. Primary fibroblasts (passage 2) were cultured until reaching 80% confluency.

### UHPLC-Q-Orbitrap-HRMS

Serum sample extraction was based on the validated plasma protocol of De Paepe et al.^13^, and was performed as follows: 150 µL serum sample was extracted with a mixture of 600 µL acetone/ethanol/ACN (1:1:1) and supplemented with 8 µL internal standard (25 ng/µL d-valine-d8). After vortexing and protein precipitation (30 min, 4 °C), followed by centrifugation (4 min, 15,000×*g*, 4 °C), the supernatant was evaporated to dryness using a GyroVap centrifugal evaporator (35 °C, vacuum conditions) (HOWE, Banbury, UK). The residue was reconstituted by addition of 200 µL ultrapure water and transferred to a glass HPLC-vial.

Cell lysates were extracted following Rombouts et al.^28^. Briefly, medium was aspirated and stored for supernatant analysis. Cells were washed with 0.9% NaCl on ice and a 1 mL 50:50 (%v/v) methanol:ultrapure water mixture was added along with 27 µL of internal standard (25 ng µL^−1^ d-valine-d8), after which the cells were scraped and transferred to Eppendorf tubes for sonication of the cells. After spin-down of the cell debris, 100 µL of the lysate was vacuum dried and dissolved in 1.5 mL of a solvent mixture. Generic extraction of the fibroblast supernatant was performed according to Wang et al.^29^. As such, the supernatant was centrifuged (15 min at 15,000×*g*) and diluted with a 50:50 (%v/v) methanol:ultrapure water extraction solvent mixture in a ratio of 2 parts supernatant to 8 parts extraction solvent, after which 3 µL of internal standard (25 ng/µL of d-valine-d_8_) was added. The extract mixture was then vacuum dried as previously described and reconstituted in 100 µL of ultrapure water.

### Method validation

Precision parameters including instrumental, intra- and inter-day precision for both targeted and untargeted analysis were evaluated. The parameters were evaluated based on the CV performance. Instrumental precision was measured by repeated injection (n = 10) of a QC sample. Intra-assay precision was assessed by extracting multiple QC samples (n = 10) in parallel, with inter-day precision including the laboratory variation introduced by extractions (n = 20) performed by different analysts on different days^30^. The Food and Drug Administration recommends a CV of 20% for tests operating near the limit of detection with targeted approaches, while a CV of 30% is considered acceptable for untargeted fingerprinting, as ions with higher CVs are not considered as potential biomarkers^31,32^.

### Reagents and chemicals

Internal (d-valine-d8) and analytical standards (Supplementary Table 1) were purchased from ICN Biomedicals (Ohio, USA), TLC Pharmchem (Vaughan, Ontario, Canada), Sigma-Aldrich (St. Louis, Missouri, USA), and Cambridge Isotope Laboratories (Tewksbury, Massachusetts, USA), respectively. Solvents were purchased from Fisher Scientific (Loughborough, UK) and VWR (Merck, Darmstadt, Germany). Ultrapure water was obtained using a water purification system from Millipore (Brussels, Belgium).

### Instrumentation

Ultra-high performance liquid chromatography coupled to high resolution mass spectrometry (UHPLC-HRMS) analysis was based on the in-house developed protocol described by Vanden Bussche et al.^33^ Chromatographic separation was performed on a Dionex UltiMate 3000 XRS UHPLC system (Thermo Fisher Scientific, San José, CA, USA) using an Acquity HSS T3 C18 column (1.8 mm, 150 × 2.1 mm) (Waters, Manchester, UK) conditioned at a set temperature of 45 °C. The solvent system was run at a fixed flow rate of 400 µL min^−1^ and consisted of ultrapure water (A) and acetonitrile (B), both acidified with 0.1% formic acid. For the separation, a gradient flow was used with the following percentages (v/v) of solvent A: 0–1.5 min at 98%, 1.5–7.0 min from 98 to 75%, 7.0–8.0 min from 75 to 40%, 8.0–12.0 min from 40 to 5%, 12.0–14.0 min at 5%, 14.0–14.1 min from 5 to 98%, followed by 4.0 min of re-equilibration to 98%. Samples were injected per volume of 10 µL. After injection, the sample was ionized for both polarities by heated electrospray ionization (HESI-II source). Following ionization, detection was performed on the Q-Exactive™ stand-alone quadrupole Orbitrap high-resolution bench-top mass spectrometer (Thermo Fisher Scientific, San José, CA, USA). Instrument settings were auxiliary, sheath and sweep gas flow rates of 25, 50, and 3 a.u.; capillary and heather temperature of 250 °C and 350 °C; a spray voltage of ± 4.0 kV; an S-lens RF level of 50 V; an *m/z* scan range of 53.4 to 800 Da; and an automatic gain control target of 1e^6^ ions. The mass resolution and maximum injection time settings were 140,000 full width at half maximum and 70 ms. The mass spectrometer was calibrated prior to use by injecting ready-to-use calibration mixtures (Thermo Fisher Scientific, San José, CA, USA) to ensure proper instrument calibration. The first and last sample injected for analysis was a standard mixture of 144 target analytes plus internal standard (Supplementary Table 1), at a concentration of 5 ng µL^−1^ to verify the correct instrument operating parameter settings and for possible compound identification. Since instrumental variation can occur during the run, quality control (QC) samples (a pool of all test samples analyzed) were included at the beginning of the analysis to stabilize the column and instrument and to check for signal deviation after every 10 samples analyzed.

### Data analysis

Xcalibur 3.0 software (Thermo Fisher Scientific, San José, CA, USA) was used for the targeted analysis of the data, with compounds identified by *m/z*-values, retention time, and C-isotope profile. Univariate statistical analysis of the serum samples on the QC-normalized targeted data was performed through R version 3.4.3 integrated into our in-house data analysis pipeline (https://github.com/UGent-LIMET). Model validity of the pairwise comparisons was evaluated by Wilcoxon rank-sum test or Welsh Two-Sample t-testing for non-parametric and parametric data, respectively.

For untargeted data analysis, automated peak extraction, peak alignment, noise removal and deconvolution were performed using Compound Discoverer™ (Thermo Fisher Scientific, San José, CA, USA). The key parameter settings for peak extraction were a retention time width of 0.3 min, a minimum peak intensity of 500,000 a.u., and a mass window of 6 ppm. The output of cell lysates and supernatant was normalized by internal standard to account for cell harvest efficiency. Prior to univariate statistical analysis, all untargeted metabolomics data were normalized by total-ion current, log transformed, and Pareto scaled to induce normality. Simca 17 (Umetrics AB, Umea, Sweden) was used for multivariate statistical analysis on serum samples. This was initiated by Principal Component Analysis for exploration of the data, which allowed for the removal of outliers (via Hotelling’s T^2^ test) and the search for unbiased clustering of samples. Following these first initial explorations, Orthogonal Partial Least Squares Discriminant Analysis (OPLS-DA) models were built to test the predictive power of the and robustness of the components included in the model, which can be validated using the accompanying quality parameters, being R^2^(Y), Q^2^(Y), a validated permutation test (n = 100) and a significant CV-ANOVA test, respectively. For untargeted discriminative component selection of serum metabolomics, volcano plots were used with the following cut-off values: fold change >|2|, p-value < 0.05. Univariate statistical analysis of the untargeted data of the cell supernatant and lysates was again performed on pareto scaled and log transformed data, after which differentiating components between groups, with a t-test p-value < 0.05, were retained.

qPCR data were analyzed by paired t-tests, and correlations were evaluated using the Pearson coefficient.

General statistical analysis and data visualization were performed using MetaboAnalyst 5.0 (Xia Lab, McGill University, Quebec, Canada), Prism 9 (GraphPad Software, San Diego, CA, USA), SPSS statistics 27 (IBM Corp., Armonk, NY, USA).

### RNA extraction

Total RNA was extracted using the BioRad Aurum kit (Bio-Rad Laboratories, Hercules, CA, USA) with on-column DNase I treatment. RNA concentration and purity were determined using NanoDrop technology (Eppendorf, Rotselaar, Belgium). All samples showed high purity with OD260/OD280 ratios between 1.8 and 2.2.

### Quantitative real-time PCR

One microgram of total RNA was reverse transcribed into single-stranded cDNA using the SensiFAST cDNA synthesis kit (Bioline, Cincinnati, OH, USA) according to the manufacturer’s instructions. Fifteen ng was used for quantitative real-time PCR (qRT-PCR) using SYBR Green (GC biotech, Alphen a/d Rijn, The Netherlands) and 250 nM forward and reverse primers. A two-step program was performed on a LightCycler 480 II system (Roche, Vilvoorde, Belgium). Cycling conditions were 95 °C for 10 min, 45 cycles of 95 °C for 10 s and 60 °C for 1 min. Primer specificity was confirmed by melting curve analysis. All reactions were performed in duplicate, and data were normalized to the expression of housekeeping genes *succinate dehydrogenase complex A subunit* (*SDHA*) and *hydroxymethylbilane synthase* (*HMBS*). The PCR efficiency of each primer pair was evaluated using a reference cDNA standard curve. Amplification efficiency was determined using the formula 10^–1^^/slope^, and primer pairs were selected based on an efficiency of 90–110%. Expression levels were calculated using the ΔΔCt method. The sequences of the primer pairs and their efficiencies are listed in Supplementary Table 5.

### Supplementary Information

Supplementary Information.

## Data Availability

Data is maintained at servers of Ghent University and include the .RAW files associated with each serum and supernatant samples generated from Xcalibur 3.0, Compound Discover and SIMCA-P 17.0 programs. Metadata of included patients (anonymized) and normalized real-time PCR values are available in .csv files. Data will be made available upon request via the corresponding author.
