# Supplementary material for: A comprehensive metabolite fingerprint of fibrostenosis in patients with Crohn’s disease
Source: Sci Rep. 2023 Dec 27;13:23036. doi: 10.1038/s41598-023-50461-1 (PMC10754816; doi:10.1038/s41598-023-50461-1)
Supplement: Supplementary file 1 — Supplementary Information. [file 41598_2023_50461_MOESM1_ESM.docx]

**SUPPLEMENTARY TABLES AND FIGURES**

**Supplementary Table 1.** Standards included in the standard reference mixture for polar metabolomics. Samples were left out for targeted analysis when they had a CV < 30% in the QC samples, or if they had > 20% missing data.

| **Metabolite** | **Chemical  formula** | **Main ion** | ***m/z*-value** | **RT (min)** | **Targeted analysis** |
| --- | --- | --- | --- | --- | --- |
| 2,4-Di-tert-butylphenol | C_14_H_20_O | [M+H]^+^ | 205.15869 | 12.9 | Yes |
| Glycerol | C_3_H_8_O_3_ | [M+H]^+^ | 93.05462 | 1.0 | No |
| 1,3-Propanediol | C_3_H_8_O_2_ | [M+H]^+^ | 77.05971 | 1.2 | Yes |
| Glucitol (Sorbitol) | C_6_H_14_O_6_ | [M+H]^+^ | 183.08631 | 1.0 | No |
| Mannitol | C_6_H_14_O_6_ | [M+H]^+^ | 183.08631 | 0.9 | No |
| 4-Methylcatechol | C_7_H_8_O_2_ | [M+H]^+^ | 125.05971 | 8.4 | Yes |
| Caffeic acid | C_9_H_8_O_4_ | [M-H]^-^ | 179.03498 | 8.8 | No |
| 3,4-Dihydroxyphenylacetic acid | C_8_H_8_O_4_ | [M+H]^+^ | 169.04954 | 8.7 | No |
| D-Gluconic acid sodium salt | C_6_H_11_NaO_7_ | [M+H]^+^ | 219.04752 | 1.1 | Yes |
| Gentisic acid | C_7_H_6_O_4_ | [M+H]^+^ | 155.03389 | 6.3 | No |
| 4-Hydroxyphenylacetic acid | C_8_H_8_O_3_ | [M-H]^-^ | 151.04007 | 9.5 | Yes |
| 2-Hydroxyphenylacetic acid | C_8_H_8_O_3_ | [M+H]^+^ | 153.05462 | 9.5 | Yes |
| 3-Hydroxyphenylacetic acid | C_8_H_8_O_3_ | [M+H]^+^ | 153.05462 | 10.5 | Yes |
| m-Coumaric acid | C_9_H_8_O_3_ | [M+H]^+^ | 165.05462 | 10.5 | Yes |
| Salicylic acid | C_7_H_6_O_3_ | [M+H]^+^ | 139.03897 | 7.8 | Yes |
| Oxoglutaric acid | C_5_H_6_O_5_ | [M+H]^+^ | 147.02880 | 1.6 | Yes |
| beta-Hydroxyisovaleric acid | C_5_H_10_O_3_ | [M+H]^+^ | 119.07027 | 4.6 | Yes |
| Syringic acid | C_9_H_10_O_5_ | [M+H]^+^ | 199.06010 | 9.0 | Yes |
| Vanillic acid | C_8_H_8_O_4_ | [M+H]^+^ | 169.04954 | 8.7 | No |
| Mesalazine | C_7_H_7_NO_3_ | [M+H]^+^ | 154.04987 | 1.5 | No |
| 3-Phenylpropionic acid | C_9_H_10_O_2_ | [M-H]^-^ | 149.06080 | 9.5 | Yes |
| Valeric acid | C_5_H_10_O_2_ | [M+H]^+^ | 103.07536 | 10.3 | Yes |
| Hexanoic acid | C_6_H_12_O_2_ | [M+H]^+^ | 117.09101 | 11.8 | Yes |
| Cyclohexanecarboxylic acid | C_7_H_12_O_2_ | [M+H]^+^ | 129.09101 | 10.0 | No |
| Pipecolic acid | C_6_H_11_NO_2_ | [M+H]^+^ | 130.08626 | 1.5 | Yes |
| Dodecanoic acid | C_12_H_24_O_2_ | [M-H]^-^ | 199.17035 | 11.5 | Yes |
| Heptanoic acid | C_7_H_14_O_2_ | [M+H]^+^ | 131.10666 | 11.7 | No |
| 5Z,8Z,11Z,14Z,17Z-Eicosapentaenoic acid (EPA) | C_20_H_30_O_2_ | [M+H]^+^ | 303.23186 | 14.9 | No |
| Linolenic acid | C_18_H_30_O_2_ | [M+H]^+^ | 279.23186 | 13.3 | No |
| 3-Hydroxybutyric acid | C_4_H_8_O_3_ | [M+H]^+^ | 105.05462 | 2.2 | Yes |
| Lactic acid | C_3_H_6_O_3_ | [M+H]^+^ | 91.03897 | 1.0 | No |
| Methylsuccinic acid | C_5_H_8_O_4_ | [M+H]^+^ | 133.04954 | 1.0 | No |
| Adipic acid | C_6_H_10_O_4_ | [M+H]^+^ | 145.05063 | 6.9 | Yes |
| Glutaric acid | C_5_H_8_O_4_ | [M+H]^+^ | 133.04954 | 5.4 | No |
| Sebacic acid | C_10_H_18_O_4_ | [M-H]^-^ | 201.11323 | 11.0 | Yes |
| Dodecanedioic acid | C_12_H_22_O_4_ | [M-H]^-^ | 229.14453 | 11.5 | No |
| Pimelic acid | C_7_H_12_O_4_ | [M+H]^+^ | 161.08084 | 8.5 | Yes |
| Citric acid | C_6_H_8_O_7_ | [M+H]^+^ | 193.03428 | 1.5 | Yes |
| Urocanic acid | C_6_H_6_N_2_O_2_ | [M+H]^+^ | 139.05020 | 1.5 | No |
| Imidazolepropionic acid | C_6_H_8_N_2_O_2_ | [M+H]^+^ | 141.06585 | 1.3 | No |
| gamma-Caprolactone | C_6_H_10_O_2_ | [M+H]^+^ | 115.07536 | 9.6 | No |
| 1,3-Dimethoxybenzene | C_8_H_10_O_2_ | [M+H]^+^ | 139.07536 | 11.9 | Yes |
| Dipropyl disulfide | C_6_H_14_S_2_ | [M+H]^+^ | 151.06097 | 1.5 | Yes |
| D-Fructose | C_6_H_12_O_6_ | [M-H]^-^ | 179.05611 | 1.1 | No |
| D-Galactose | C_6_H_12_O_6_ | [M-H]^-^ | 179.05611 | 1.0 | No |
| D-Glucose | C_6_H_12_O_6_ | [M+Na]^+^ | 203.05261 | 1.0 | Yes |
| D-Gluconic acid | C_6_H_12_O_7_ | [M-H]^-^ | 195.05103 | 1.0 | Yes |
| 1-Octen-3-one | C_8_H_14_O | [M+H]^+^ | 127.11174 | 12.1 | Yes |
| 2,3-Heptanedione | C_7_H_12_O_2_ | [M+H]^+^ | 129.09101 | 11.1 | Yes |
| 2,3-Pentanedione | C_5_H_8_O_2_ | [M+H]^+^ | 101.05971 | 1.1 | Yes |
| 2-Octanone/ 3-Octanone | C_8_H_16_O | [M+H]^+^ | 129.12739 | 12.7 | Yes |
| 3-Heptanone | C_7_H_14_O | [M+H]^+^ | 115.11174 | 12.1 | No |
| Acetophenone | C_8_H_8_O | [M+H]^+^ | 121.06479 | 11.3 | Yes |
| Cyclohexanone | C_6_H_10_O | [M+H]^+^ | 99.08044 | 10.9 | No |
| L-Alanine | C_3_H_7_NO_2_ | [M+H]^+^ | 90.05496 | 1.0 | No |
| L-Valine | C_5_H_11_NO_2_ | [M+H]^+^ | 118.08626 | 1.1 | Yes |
| L-Leucine | C_6_H_13_NO_2_ | [M+H]^+^ | 132.10191 | 2.5 | Yes |
| L-Isoleucine | C_6_H_13_NO_2_ | [M+H]^+^ | 132.10191 | 2.2 | Yes |
| L-Proline | C_5_H_9_NO_2_ | [M+H]^+^ | 116.07061 | 1.0 | Yes |
| L-Serine | C_3_H_7_NO_3_ | [M+H]^+^ | 106.04987 | 0.9 | Yes |
| L-Threonine | C_4_H_9_NO_3_ | [M+H]^+^ | 120.06552 | 1.0 | Yes |
| L-Aspartic acid | C_4_H_7_NO_4_ | [M+H]^+^ | 134.04478 | 0.9 | Yes |
| L-Methionine | C_5_H_11_NO_2_S | [M+H]^+^ | 150.05833 | 1.5 | Yes |
| L-Pyroglutamic acid | C_5_H_7_NO_3_ | [M+H]^+^ | 130.04987 | 1.8 | Yes |
| L-Glutamic acid | C_5_H_9_NO_4_ | [M+H]^+^ | 148.06043 | 1.0 | Yes |
| L-Tyrosine | C_9_H_11_NO_3_ | [M+H]^+^ | 182.08117 | 2.0 | Yes |
|  |  |  |  |  |  |
| gamma-Aminobutyric acid | C_4_H_9_NO_2_ | [M+H]^+^ | 104.07061 | 1.0 | Yes |
| 5-Aminovaleric acid | C_5_H_11_NO_2_ | [M+H]^+^ | 118.08626 | 1.1 | No |
| Thiazolidine-2-carboxylic acid | C_4_H_7_NO_2_S | [M+H]^+^ | 134.02703 | 1.1 | Yes |
| L-Ornithine | C_5_H_12_N_2_O_2_ | [M-H]^-^ | 131.08260 | 0.8 | Yes |
| L-Lysine | C_6_H_14_N_2_O_2_ | [M+H]^+^ | 147.11280 | 0.9 | Yes |
| 2-Amino(iso)butyric acid | C_4_H_9_NO_2_ | [M+H]^+^ | 104.07061 | 1.0 | Yes |
| D-Aspartic acid | C_4_H_7_NO_4_ | [M+H]^+^ | 134.04478 | 1.0 | Yes |
| D-Glutamic acid | C_5_H_9_NO_4_ | [M+H]^+^ | 148.06043 | 1.0 | Yes |
| L-Arginine | C_6_H_14_N_4_O_2_ | [M+H]^+^ | 175.11895 | 0.9 | Yes |
| L-Asparagine | C_4_H_8_N_2_O_3_ | [M+H]^+^ | 133.06077 | 0.9 | No |
| L-Histidine | C_6_H_9_N_3_O_2_ | [M+H]^+^ | 156.07675 | 0.9 | Yes |
| 3-Indoleacetic acid | C_10_H_9_NO_2_ | [M+H]^+^ | 176.07061 | 10.8 | Yes |
| N-Acetylglutamic acid | C_7_H_11_NO_5_ | [M+H]^+^ | 188.05645 | 1.8 | No |
| Nicotinic acid | C_6_H_5_NO_2_ | [M+H]^+^ | 124.03930 | 1.5 | Yes |
| Pyrrole-2-carboxylic acid | C_5_H_5_NO_2_ | [M+H]^+^ | 112.03930 | 5.9 | Yes |
| L-Glutamine | C_5_H_10_N_2_O_3_ | [M+H]^+^ | 147.07642 | 0.9 | Yes |
| N-Acetyl-L-methionine | C_7_H_13_NO_3_S | [M+H]^+^ | 192.06889 | 7.6 | Yes |
| Acetylcarnitine | C_9_H_17_NO_4_ | [M+H]^+^ | 204.12303 | 1.5 | Yes |
| 3-Methyl-2-cyclohexen-1-on | C_7_H_10_O | [M+H]^+^ | 111.08044 | 10.8 | Yes |
| Isoamyl propionate | C_8_H_16_O_2_ | [M+H]^+^ | 145.12231 | 10.9 | No |
| Oleoyl-L-carnitine | C_25_H_47_NO_4_ | [M+H]^+^ | 426.35779 | 13.2 | Yes |
| Methional | C_4_H_8_OS | [M+H]^+^ | 105.03686 | 6.9 | No |
| trans-4-Hydroxy-L-proline | C_5_H_9_NO_3_ | [M+H]^+^ | 132.06552 | 0.9 | Yes |
| 2-Piperidinone | C_5_H_9_NO | [M+H]^+^ | 100.07569 | 5.0 | Yes |
| Spermine | C_10_H_26_N_4_ | [M+H]^+^ | 203.22302 | 0.7 | Yes |
| Dopamine | C_8_H_12_O_2_Cl | [M+NH4]^+^ | 193.08641 | 1.5 | No |
| Spermidine | C_7_H_19_N_3_ | [M+H]^+^ | 146.16517 | 0.7 | Yes |
| Cyclohexylamine | C_6_H_13_N | [M+H]^+^ | 100.11208 | 4.1 | Yes |
| 2-(Dimethylamino)acetonitrile | C_4_H_8_N_2_ | [M+H]^+^ | 85.07602 | 1.0 | No |
| 3-Hydroxypyridine | C_5_H_5_NO | [M+H]^+^ | 96.04439 | 1.0 | No |
| Cytosine | C_4_H_5_N_3_O | [M+H]^+^ | 112.05054 | 1.0 | No |
| Hypoxanthine | C_5_H_4_N_4_O | [M+H]^+^ | 137.04579 | 1.5 | Yes |
| Taurocholic acid | C_26_H_45_NO_7_S | [M+H]^+^ | 516.29895 | 9.8 | Yes |
| Thiabendazole | C_10_H_7_N_3_S | [M+H]^+^ | 202.04334 | 8.5 | Yes |
| Thymine | C_5_H_6_N_2_O_2_ | [M+H]^+^ | 127.05020 | 1.6 | Yes |
| Allantoin | C_4_H_6_N_4_O_3_ | [M+H]^+^ | 159.05127 | 1.0 | Yes |
| Crotonaldehyde | C_4_H_6_O | [M+H]^+^ | 71.04914 | 6.2 | No |
| Phenylacetaldehyde | C_8_H_8_O | [M+H]^+^ | 121.06479 | 11.0 | Yes |
| Cinnamaldehyde | C_9_H_8_O | [M+H]^+^ | 133.06479 | 11.6 | No |
| Propanal | C_3_H_6_O | [M+H]^+^ | 59.04914 | 4.4 | Yes |
| Salicylaldehyde | C_7_H_6_O_2_ | [M+H]^+^ | 123.04406 | 9.7 | Yes |
| 2-Ethyl-1-hexene | C_8_H_16_ | [M+H]^+^ | 113.13248 | 11.1 | No |
| beta-Pinene | C_10_H_16_ | [M+H]^+^ | 137.13248 | 10.8 | Yes |
| Styrene | C_8_H_8_ | [M+H]^+^ | 105.06988 | 11.1 | Yes |
| Cholic acid | C_24_H_40_O_5_ | [M-H]^-^ | 407.28030 | 12.0 | Yes |
| Sodium taurocholate (TCA) | C_26_H_44_NNaO_7_S | [M-H]^-^ | 514.28440 | 11.3 | Yes |
| Glycolithocholic acid (GLCA) | C_26_H_43_NO_4_ | [M-H]^-^ | 432.31193 | 13.0 | Yes |
| Sodium taurodeoxycholate hydrate (TDCA)/ Tauroursodeoxycholic acid (TUDCA) | C_26_H_44_NO_6_SNa/ C_26_H_45_NO_6_S | [M-Na]^-^ | 498.28948 | 14.28 | Yes |
| Sodium glycodeoxycholate (GDCA)/ Glycoursodeoxycholic acid (GUDCA)/ Sodium glycochenodeoxycholate (GCDCA) | C_26_H_42_NO_5_Na/ C_26_H_43_NO_5_/ C_26_H_42_NNaO_5_ | [M-H]^-^ | 448.30685 | 12.2 | Yes |
| Chenodeoxycholic acid (CDCA)/ Deoxycholic acid (DCA)/ Ursodeoxycholic acid (UDCA) | C_24_H_40_O_4_ | [M-H]^-^ | 391.28534 | 13.1 | Yes |
| Cortisol | C_21_H_30_O_5_ | [M+H]^+^ | 363.21660 | 11.1 | Yes |
| Cortisone | C_21_H_28_O_5_ | [M+H]^+^ | 361.20095 | 11.1 | Yes |
| Histamine | C_5_H_9_N_3_ | [M+H]^+^ | 112.08692 | 0.8 | No |
| Creatine | C_4_H_9_N_3_O_2_ | [M+H]^+^ | 132.07680 | 0.9 | Yes |
| Creatinine | C_4_H_7_N_3_O | [M+H]^+^ | 114.06610 | 0.9 | Yes |
| 1-Methylhistidine | C_7_H_11_N_3_O_2_ | [M+H]^+^ | 170.09240 | 0.9 | Yes |
| Taurine | C_2_H_7_NO_3_S | [M+H]^+^ | 126.02190 | 0.9 | No |
| L-carnitine | C_7_H_15_NO_3_ | [M+H]^+^ | 162.11250 | 1.0 | Yes |
| Mannose | C_6_H_12_O_6_ | [M+Na]^+^ | 203.05261 | 7.5 | Yes |
| Betaine | C_5_H_11_NO_2_S | [M+H]^+^ | 118.08626 | 1.0 | Yes |
| Xylose | C_5_H_10_O_5_ | [M+H]^+^ | 151.06010 | 1.0 | Yes |
| Acetylcholine chloride | C_7_H_16_ClNO_2_ | [M+H]^+^ | 146.11756 | 1.3 | Yes |
| Choline | C_5_H_14_NO.Cl | [M+H]^+^ | 104.07061 | 1.0 | Yes |
| Serotonine | C_10_H_12_NO_2_ | [M+H]^+^ | 177.10224 | 3.8 | Yes |
| 3-Methoxytyramine | C_9_H_13_NO_2_ | [M+H]^+^ | 168.10191 | 4.4 | No |
| Kynurenine | C_10_H_12_N_2_O_3_ | [M+H]^+^ | 209.09207 | 4.6 | Yes |
| 2-Phenylethylamine | C_8_H_11_N | [M+H]^+^ | 122.09643 | 6.6 | Yes |
| 2-Methylglutaric acid | C_6_H_10_O_4_ | [M+H]^+^ | 147.06519 | 7.2 | Yes |
| Tryptophan | C_11_H_12_N_2_O_2_ | [M+H]^+^ | 205.09715 | 7.5 | Yes |
| 5-Hydroxyindole acetic acid | C_10_H_9_NO_3_ | [M+H]^+^ | 192.06552 | 8.4 | Yes |
| 4-Methyl-2-oxovaleric acid | C_6_H_10_O_3_ | [M+H]^+^ | 131.07021 | 8.4 | Yes |
| 3-Methylbutyryl carnitine | C_12_H_23_NO_4_ | [M+H]^+^ | 246.16998 | 8.6 | Yes |
| 2-Hydroxyisocaproic acid | C_6_H_12_O_3_ | [M+H]^+^ | 133.08592 | 9.3 | Yes |
| DL-hexanoylcarnitine chloride | C_13_H_25_ClNO_4_ | [M+H]^+^ | 260.18563 | 10.3 | Yes |
| Corticosterone | C_21_H_30_O_4_ | [M+H]^+^ | 347.22170 | 11.5 | Yes |
| Testosteron | C_19_H_28_O_2_ | [M+H]^+^ | 289.21621 | 12.5 | No |
| Lysophosphatidylcholine_C14:0 | C_22_H_46_NO_7_P | [M+H]^+^ | 468.30847 | 12.5 | Yes |
| Lysophosphatidylcholine_C18:1 | C_26_H_52_NO_7_P | [M+H]^+^ | 522.35542 | 13.7 | Yes |
|  |  |  |  |  |  |
| *Internal standard* |  |  |  |  |  |
| D-Valine-d8 | C_5_H_3_O_2_D_8_N | [M+H]^+^ | 126.13647 | 1.3 | IS |

RT, retention time; IS: internal standard.

**Supplementary Table 2**. Patient characteristics

|  | **Stenotic** | **Non-stenotic** | **p-value** |
| --- | --- | --- | --- |
| N | 28 | 38 |  |
| Age at (yrs; median, range): |  |  |  |
| Crohn's disease diagnosis | 26 (16-70) | 27 (14-54) | NS |
| Stenosis diagnosis | 27 (16-72) | NA | NA |
| Gender (% male) | 50.0% | 44,7% | NS |
| Anti-TNF exposure | 9 (32.1%) | 5 (13.2%) | NS |
| Montreal classification: |  |  | NA |
| L1 (ileal) | 21 (75.0%) | 24 (63.2%) | NS |
| L3 (ileocolonic) | 7 (25.0%) | 14 (36.8%) | NS |
| Time to stenosis (yrs; median, range) | 1 (0-10) | NA | NA |
| Crohn’s disease status (symptomatic) | 19 (67.9%) | 21 (55.3%) | NS |

Anti-TNF: anti-tumor necrosis factor; NS: not significant; NA: not applicable. Mann-Whitney U and Chi-square test were applied depending on the distribution and nature of the data.

**Supplementary Table 3**. Untargeted components differentially abundant between stenotic and non-stenotic patient sera

| Component ID | *m/z* | RT (min) | Ionisation | FC | log2(FC) | raw.pval | -log10(p) |
| --- | --- | --- | --- | --- | --- | --- | --- |
| 117 | 365.10486 | 1.2 | [M+H]^+^ | 0.025 | -5.30 | 3.49e^-3^ | 2.46 |
| 132 | 250.11166 | 11.0 | [M-H]^-^ | 145.61 | 7.18 | 1.04e^-2^ | 1.98 |
| 187 | 182.98793 | 10.7 | [M-H]^-^ | 0.058 | -4.11 | 1.43e^-2^ | 1.84 |
| 193 | 131.03392 | 4.1 | [M+H]^+^ | 4.81 | 2.26 | 8.73e^-3^ | 2.06 |
| 216 | 457.27869 | 14.9 | [M+H]^+^ | 3.94 | 1.98 | 1.82e^-2^ | 1.74 |
| 262 | 479.26050 | 14.7 | [M+H]^+^ | 4.91 | 2.29 | 2.52e^-2^ | 1.60 |
| 273 | 357.27820 | 12.0 | [M+H]^+^ | 0.19 | -2.38 | 7.54e^-3^ | 2.12 |
| 318 | 336.32578 | 13.4 | [M+H]^+^ | 0.46 | -1.11 | 1.51e^-2^ | 1.82 |
| 395 | 784.58374 | 15.7 | [M+H]^+^ | 0.50 | -1.01 | 3.98e^-2^ | 1.40 |
| 471 | 232.15405 | 6.4 | [M+H]^+^ | 0.48 | -1.05 | 1.75e^-2^ | 1.76 |
| 553 | 242.13837 | 10.5 | [M+H]^+^ | 2.07 | 1.05 | 2.59e^-2^ | 1.59 |
| 558 | 357.27802 | 12.1 | [M+H]^+^ | 0.29 | -1.77 | 2.03e^-2^ | 1.69 |
| 567 | 172.13307 | 14.5 | [M+H]^+^ | 4.09 | 2.03 | 4.99e^-2^ | 1.30 |
| 598 | 357.27826 | 12.4 | [M+H]^+^ | 0.22 | -2.19 | 4.53e^-2^ | 1.34 |
| 634 | 185.11758 | 11.4 | [M-H]^-^ | 2.28 | 1.19 | 4.94e^-2^ | 1.31 |
| 651 | 228.99379 | 10.7 | [M-H]^-^ | 0.057 | -4.13 | 1.47e^-2^ | 1.83 |
| 658 | 266.21109 | 11.4 | [M+H]^+^ | 2.03 | 1.02 | 2.02e^-2^ | 1.69 |
| 731 | 375.28854 | 12.0 | [M+H]^+^ | 0.19 | -2.40 | 5.49e^-3^ | 2.26 |
| 756 | 265.14792 | 11.2 | [M-H]^-^ | 0.39 | -1.34 | 1.22e^-2^ | 1.91 |
| 761 | 183.03352 | 8.2 | [M+H]^+^ | 0.21 | -2.26 | 7.90e^-3^ | 2.10 |
| 772 | 729.58887 | 16.0 | [M+H]^+^ | 0.50 | -1.00 | 4.98e^-3^ | 2.30 |
| 813 | 675.67493 | 16.0 | [M+H]^+^ | 0.46 | -1.11 | 2.85e^-2^ | 1.54 |
| 821 | 355.28363 | 15.1 | [M+H]^+^ | 3.47 | 1.79 | 4.42e^-2^ | 1.35 |
| 840 | 429.24112 | 11.4 | [M+H]^+^ | 2.62 | 1.39 | 3.16e^-2^ | 1.50 |
| 847 | 228.19565 | 14.9 | [M+H]^+^ | 2.13 | 1.09 | 1.16e^-2^ | 1.93 |
| 894 | 100.07619 | 2.1 | [M+H]^+^ | 0.40 | -1.30 | 5.67e^-3^ | 2.25 |
| 895 | 384.04709 | 10.7 | [M-H]^-^ | 0.052 | -4.26 | 2.73e^-2^ | 1.56 |
| 929 | 224.05585 | 7.7 | [M-H]^-^ | 0.40 | -1.30 | 1.92e^-2^ | 1.72 |
| 937 | 242.21135 | 14.8 | [M+H]^+^ | 2.10 | 1.07 | 8.92e^-3^ | 2.05 |
| 952 | 474.30518 | 14.9 | [M+H]^+^ | 4.96 | 2.31 | 3.67e^-3^ | 2.43 |
| 957 | 596.54315 | 14.0 | [M+H]^+^ | 2.10 | 1.07 | 3.07e^-2^ | 1.51 |
| 980 | 423.06305 | 1.2 | [M+H]^+^ | 0.083 | -3.59 | 3.78e^-3^ | 2.42 |
| 1038 | 471.24191 | 12.1 | [M-H]^-^ | 0.18 | -2.47 | 1.28e^-2^ | 1.89 |
| 1048 | 218.17487 | 8.4 | [M+H]^+^ | 2.14 | 1.10 | 2.42e^-2^ | 1.61 |
| 1067 | 325.18442 | 12.0 | [M-H]^-^ | 0.44 | -1.18 | 8.69e^-3^ | 2.06 |
| 1124 | 297.15314 | 11.7 | [M-H]^-^ | 0.46 | -1.13 | 2.95e^-2^ | 1.53 |
| 1146 | 437.29071 | 12.4 | [M-H]^-^ | 0.28 | -1.84 | 4.85e^-2^ | 1.31 |
| 1156 | 352.05746 | 10.8 | [M-H]^-^ | 0.045 | -4.48 | 1.15e^-2^ | 1.94 |
| 1182 | 131.03369 | 3.5 | [M-H]^-^ | 0.43 | -1.21 | 5.93e^-3^ | 2.23 |
| 1206 | 240.12033 | 8.4 | [M+H]^+^ | 2.23 | 1.15 | 1.30e^-2^ | 1.88 |
| 1227 | 273.21729 | 10.7 | [M+H]^+^ | 21.53 | 4.43 | 2.46e^-2^ | 1.61 |
| 1237 | 163.11159 | 11.8 | [M+H]^+^ | 3.34 | 1.74 | 4.22e^-2^ | 1.37 |
| 1244 | 283.08228 | 9.8 | [M-H]^-^ | 0.24 | -2.05 | 2.69e^-2^ | 1.57 |
| 1261 | 437.29095 | 12.0 | [M-H]^-^ | 0.19 | -2.38 | 6.70e^-3^ | 2.17 |
| 1274 | 471.24197 | 11.6 | [M-H]^-^ | 0.17 | -2.54 | 2.35e^-3^ | 2.63 |
| 1297 | 595.53967 | 13.2 | [M+H]^+^ | 2.15 | 1.10 | 3.52e^-2^ | 1.45 |
| 1357 | 278.14313 | 11.9 | [M-H]^-^ | 33.71 | 5.07 | 3.89e^-2^ | 1.41 |
| 1360 | 215.03799 | 11.3 | [M-H]^-^ | 2.43 | 1.28 | 4.24e^-2^ | 1.37 |
| 1390 | 583.32275 | 12.7 | [M+H]^+^ | 0.25 | -2.02 | 3.84e^-2^ | 1.41 |
| 1391 | 372.23907 | 11.3 | [M-H]^-^ | 2.03 | 1.02 | 1.12e^-2^ | 1.95 |
| 1393 | 188.12784 | 14.2 | [M+H]^+^ | 3.45 | 1.79 | 4.91e^-3^ | 2.31 |
| 1442 | 352.24506 | 14.4 | [M+H]^+^ | 2.46 | 1.30 | 7.29e^-3^ | 2.14 |
| 1500 | 327.25241 | 15.1 | [M+H]^+^ | 3.49 | 1.80 | 4.48e^-2^ | 1.35 |
| 1507 | 216.06883 | 5.9 | [M+H]^+^ | 0.038 | -4.70 | 2.40e^-2^ | 1.62 |
| 1531 | 357.21152 | 8.1 | [M+H]^+^ | 0.23 | -2.10 | 4.89e^-2^ | 1.31 |
| 1591 | 160.09669 | 6.8 | [M+H]^+^ | 2.05 | 1.03 | 4.18e^-2^ | 1.38 |
| 1592 | 474.30502 | 14.6 | [M+H]^+^ | 2.92 | 1.55 | 2.40e^-3^ | 2.62 |
| 1598 | 757.62012 | 15.9 | [M+H]^+^ | 0.43 | -1.22 | 4.23e^-2^ | 1.37 |
| 1618 | 471.24200 | 11.6 | [M-H]^-^ | 0.50 | -1.00 | 2.10e^-2^ | 1.68 |
| 1734 | 159.06522 | 6.9 | [M-H]^-^ | 0.43 | -1.20 | 2.96e^-2^ | 1.53 |
| 1736 | 204.98058 | 6.8 | [M-H]^-^ | 0.40 | -1.30 | 4.82e^-3^ | 2.32 |
| 1750 | 405.22366 | 14.8 | [M+H]^+^ | 4.12 | 2.04 | 7.14e^-4^ | 3.15 |
| 1761 | 342.22867 | 12.3 | [M-H]^-^ | 2.19 | 1.13 | 3.07e^-2^ | 1.51 |
| 1762 | 266.01791 | 10.9 | [M-H]^-^ | 0.23 | -2.10 | 2.42e^-2^ | 1.61 |
| 1813 | 425.25238 | 14.9 | [M+H]^+^ | 2.47 | 1.30 | 3.41e^-2^ | 1.47 |
| 1911 | 676.67798 | 15.9 | [M+H]^+^ | 0.40 | -1.32 | 2.23e^-2^ | 1.65 |
| 1934 | 114.05522 | 5.4 | [M+H]^+^ | 0.29 | -1.79 | 9.77e^-3^ | 2.01 |
| 1966 | 230.13855 | 8.8 | [M+H]^+^ | 2.03 | 1.02 | 1.54e^-2^ | 1.81 |
| 1969 | 617.52167 | 13.2 | [M+H]^+^ | 2.26 | 1.17 | 3.35e^-2^ | 1.47 |
| 2059 | 329.08783 | 6.3 | [M-H]^-^ | 0.39 | -1.35 | 4.17e^-2^ | 1.38 |
| 2072 | 254.13589 | 9.8 | [M+H]^+^ | 2.41 | 1.27 | 3.12e^-2^ | 1.50 |
| 2141 | 160.09676 | 6.3 | [M+H]^+^ | 0.44 | -1.19 | 5.80e^-3^ | 2.24 |
| 2146 | 471.24213 | 11.7 | [M-H]^-^ | 0.39 | -1.34 | 1.85e^-2^ | 1.73 |
| 2203 | 232.99449 | 10.7 | [M-H]^-^ | 0.22 | -2.17 | 4.43e^-2^ | 1.35 |
| 2233 | 633.51654 | 13.3 | [M+H]^+^ | 2.27 | 1.18 | 3.62e^-2^ | 1.44 |
| 2273 | 744.58856 | 15.3 | [M+H]^+^ | 0.49 | -1.01 | 8.82e^-3^ | 2.05 |
| 2313 | 455.24734 | 13.3 | [M-H]^-^ | 0.47 | -1.08 | 2.85e^-2^ | 1.54 |
| 2334 | 381.07852 | 1.2 | [M+H]^+^ | 0.26 | -1.91 | 1.34e^-3^ | 2.87 |
| 2409 | 187.14397 | 7.1 | [M+H]^+^ | 0.34 | -1.56 | 1.44e^-3^ | 2.85 |
| 2417 | 259.00995 | 10.9 | [M-H]^-^ | 0.37 | -1.41 | 2.75e^-2^ | 1.56 |
| 2431 | 405.22388 | 14.6 | [M+H]^+^ | 2.54 | 1.35 | 3.97e^-3^ | 2.40 |
| 2450 | 463.37723 | 13.0 | [M+H]^+^ | 0.41 | -1.29 | 2.63e^-2^ | 1.58 |
| 2564 | 336.12360 | 8.6 | [M-H]^-^ | 2.36 | 1.24 | 4.29e^-2^ | 1.37 |
| 2568 | 269.06671 | 8.1 | [M-H]^-^ | 0.17 | -2.52 | 4.35e^-3^ | 2.36 |
| 2570 | 100.07610 | 6.3 | [M+H]^+^ | 0.33 | -1.59 | 2.71e^-3^ | 2.57 |
| 2599 | 471.24203 | 12.0 | [M-H]^-^ | 0.13 | -2.90 | 1.96e^-2^ | 1.71 |
| 2636 | 258.04407 | 3.1 | [M-H]^-^ | 2.34 | 1.23 | 2.86e^-2^ | 1.54 |
| 2646 | 214.03448 | 6.2 | [M+H]^+^ | 0.44 | -1.19 | 2.53e^-3^ | 2.60 |
| 2648 | 425.36365 | 12.9 | [M-H]^-^ | 0.48 | -1.04 | 1.82e^-3^ | 2.74 |
| 2660 | 438.29422 | 12.0 | [M-H]^-^ | 0.25 | -2.00 | 6.18e^-3^ | 2.21 |
| 2686 | 273.05927 | 2.8 | [M-H]^-^ | 5.04 | 2.33 | 2.26e^-2^ | 1.64 |
| 2690 | 158.02705 | 6.1 | [M+H]^+^ | 0.17 | -2.55 | 1.11e^-2^ | 1.95 |
| 2765 | 528.26385 | 11.1 | [M-H]^-^ | 0.33 | -1.59 | 1.72e^-2^ | 1.76 |
| 2801 | 258.20615 | 10.9 | [M+H]^+^ | 2.04 | 1.03 | 1.56e^-2^ | 1.80 |
| 2937 | 696.41479 | 11.8 | [M-H]^-^ | 2.31 | 1.21 | 6.00e^-3^ | 2.22 |
| 2976 | 371.24191 | 14.7 | [M+H]^+^ | 2.18 | 1.12 | 6.48e^-3^ | 2.19 |
| 2985 | 214.17993 | 14.9 | [M+H]^+^ | 2.06 | 1.04 | 1.54e^-2^ | 1.81 |
| 2991 | 240.12381 | 10.2 | [M-H]^-^ | 2.17 | 1.12 | 1.02e^-2^ | 1.99 |
| 3037 | 512.40344 | 15.5 | [M-H]^-^ | 2.17 | 1.12 | 4.01e^-3^ | 2.40 |
| 3046 | 393.22379 | 14.4 | [M+H]^+^ | 2.44 | 1.29 | 8.51e^-3^ | 2.07 |
| 3061 | 201.15965 | 8.1 | [M+H]^+^ | 0.31 | -1.69 | 9.43e^-3^ | 2.02 |
| 3146 | 366.22452 | 11.7 | [M+H]^+^ | 2.07 | 1.05 | 4.21e^-2^ | 1.37 |
| 3154 | 471.24237 | 11.8 | [M-H]^-^ | 0.25 | -1.97 | 2.71e^-3^ | 2.57 |
| 3168 | 134.06012 | 6.4 | [M+H]^+^ | 0.39 | -1.36 | 6.43e^-3^ | 2.19 |
| 3220 | 169.04933 | 6.5 | [M+H]^+^ | 0.48 | -1.05 | 1.57e^-3^ | 2.80 |
| 3254 | 298.15643 | 11.6 | [M-H]^-^ | 0.46 | -1.11 | 1.50e^-2^ | 1.82 |
| 3261 | 446.99393 | 8.5 | [M-H]^-^ | 0.43 | -1.20 | 1.11e^-2^ | 1.97 |
| 3295 | 269.17596 | 13.1 | [M-H]^-^ | 2.21 | 1.14 | 4.33e^-3^ | 2.36 |
| 3305 | 455.24731 | 13.2 | [M-H]^-^ | 0.32 | -1.64 | 2.21e^-2^ | 1.65 |
| 3334 | 366.22452 | 11.4 | [M+H]^+^ | 2.13 | 1.09 | 1.11e^-2^ | 1.96 |
| 3353 | 295.13159 | 11.4 | [M-H]^-^ | 0.46 | -1.12 | 4.42e^-2^ | 1.35 |
| 3363 | 315.22748 | 9.2 | [M+H]^+^ | 2.17 | 1.12 | 3.34e^-2^ | 1.45 |
| 3380 | 150.07741 | 1.7 | [M+H]^+^ | 0.40 | -1.30 | 3.47e^-2^ | 1.46 |
| 3383 | 331.28357 | 15.7 | [M+H]^+^ | 0.48 | -1.06 | 6.31e^-3^ | 2.20 |
| 3405 | 192.06599 | 9.3 | [M-H]^-^ | 0.43 | -1.23 | 2.54e^-4^ | 3.59 |
| 3497 | 158.08134 | 6.3 | [M-H]^-^ | 0.45 | -1.14 | 2.25e^-3^ | 2.65 |
| 3519 | 553.29724 | 14.6 | [M+H]^+^ | 3.14 | 1.65 | 3.25e^-3^ | 2.49 |
| 3528 | 338.89243 | 8.6 | [M-H]^-^ | 0.41 | -1.29 | 1.61e^-2^ | 1.79 |
| 3561 | 511.40009 | 15.3 | [M-H]^-^ | 2.12 | 1.08 | 2.08e^-2^ | 1.68 |
| 3583 | 333.83286 | 7.6 | [M-H]^-^ | 0.29 | -1.80 | 1.64e^-2^ | 1.78 |
| 3648 | 448.30472 | 11.8 | [M+H]^+^ | 0.44 | -1.17 | 9.41e^-3^ | 2.03 |
| 3657 | 481.27661 | 14.9 | [M+H]^+^ | 3.18 | 1.67 | 2.47e^-2^ | 1.61 |
| 3671 | 373.11411 | 10.2 | [M-H]^-^ | 5.76 | 2.52 | 7.15e^-3^ | 2.14 |
| 3782 | 144.06541 | 4.8 | [M-H]^-^ | 0.44 | -1.16 | 2.49e^-4^ | 3.60 |
| 3809 | 621.30505 | 12.0 | [M-H]^-^ | 0.36 | -1.45 | 1.55e^-2^ | 1.81 |
| 3830 | 337.32861 | 14.7 | [M+H]^+^ | 2.05 | 1.03 | 6.44e^-3^ | 2.19 |
| 3837 | 596.49799 | 16.0 | [M-H]^-^ | 0.48 | -1.06 | 1.95e^-2^ | 1.71 |
| 3881 | 188.98543 | 3.1 | [M-H]^-^ | 0.41 | -1.27 | 1.21e^-2^ | 1.91 |
| 3918 | 355.28552 | 15.8 | [M-H]^-^ | 0.42 | -1.25 | 3.18e^-2^ | 1.50 |
| 3924 | 346.21564 | 12.0 | [M-H]^-^ | 2.13 | 1.09 | 2.64e^-2^ | 1.58 |
| 3967 | 265.09689 | 8.5 | [M+H]^+^ | 0.31 | -1.66 | 1.44e^-3^ | 2.84 |
| 4002 | 212.12808 | 9.2 | [M+H]^+^ | 3.22 | 1.69 | 1.57e^-2^ | 1.80 |
| 4008 | 453.35846 | 15.6 | [M-H]^-^ | 2.33 | 1.22 | 1.50e^-2^ | 1.82 |
| 4072 | 465.35846 | 15.1 | [M-H]^-^ | 2.25 | 1.17 | 1.88e^-2^ | 1.72 |
| 4073 | 465.35840 | 14.9 | [M-H]^-^ | 2.03 | 1.02 | 6.22e^-3^ | 2.21 |
| 4097 | 551.28149 | 14.8 | [M+H]^+^ | 3.06 | 1.61 | 1.27e^-2^ | 1.89 |
| 4112 | 295.24982 | 11.9 | [M+H]^+^ | 2.02 | 1.02 | 3.44e^-2^ | 1.46 |
| 4147 | 253.87590 | 7.6 | [M-H]^-^ | 0.26 | -1.95 | 1.29e^-2^ | 1.89 |
| 4149 | 495.29218 | 15.1 | [M+H]^+^ | 2.13 | 1.09 | 3.93e^-2^ | 1.40 |
| 4223 | 478.29385 | 12.2 | [M-H]^-^ | 0.45 | -1.16 | 7.38e^-3^ | 2.13 |
| 4258 | 238.10498 | 9.1 | [M+H]^+^ | 2.04 | 1.03 | 3.09e^-2^ | 1.51 |
| 4309 | 286.16617 | 11.1 | [M-H]^-^ | 2.03 | 1.02 | 3.33e^-2^ | 1.48 |
| 4310 | 224.10548 | 10.1 | [M-H]^-^ | 2.16 | 1.11 | 3.70e^-2^ | 1.43 |
| 4311 | 391.28571 | 11.9 | [M-H]^-^ | 0.19 | -2.41 | 1.37e^-3^ | 2.86 |
| 4329 | 170.97491 | 7.4 | [M-H]^-^ | 0.35 | -1.50 | 3.75e^-2^ | 1.42 |
| 4422 | 391.28580 | 12.5 | [M-H]^-^ | 0.29 | -1.77 | 1.73e^-3^ | 2.76 |
| 4509 | 786.58844 | 15.8 | [M+H]^+^ | 0.43 | -1.22 | 1.26e^-2^ | 1.90 |
| 4537 | 331.09128 | 8.7 | [M-H]^-^ | 0.46 | -1.12 | 4.99e^-3^ | 2.30 |
| 4613 | 373.27310 | 11.1 | [M+H]^+^ | 0.39 | -1.37 | 1.67e^-2^ | 1.78 |
| 4698 | 272.15042 | 10.4 | [M-H]^-^ | 2.17 | 1.12 | 2.12e^-2^ | 1.67 |
| 4766 | 391.28540 | 15.1 | [M-H]^-^ | 2.09 | 1.07 | 2.07e^-3^ | 2.68 |
| 4841 | 409.29590 | 14.8 | [M-H]^-^ | 2.26 | 1.17 | 1.82e^-2^ | 1.74 |
| 4946 | 678.40460 | 11.8 | [M-H]^-^ | 2.89 | 1.53 | 1.02e^-2^ | 1.99 |
| 4977 | 298.18546 | 7.8 | [M+H]^+^ | 0.34 | -1.56 | 1.93e^-2^ | 1.71 |
| 4996 | 441.35867 | 14.9 | [M-H]^-^ | 2.28 | 1.19 | 1.48e^-3^ | 2.83 |
| 5032 | 192.10515 | 7.9 | [M+H]^+^ | 0.26 | -1.91 | 6.11e^-4^ | 3.21 |
| 5294 | 377.26559 | 12.3 | [M-H]^-^ | 2.14 | 1.10 | 2.90e^-2^ | 1.54 |
| 5302 | 432.31207 | 13.1 | [M-H]^-^ | 0.32 | -1.64 | 3.15e^-2^ | 1.50 |
| 5306 | 383.22720 | 10.6 | [M+H]^+^ | 0.48 | -1.06 | 4.08e^-2^ | 1.39 |
| 5351 | 296.11234 | 7.4 | [M+H]^+^ | 2.02 | 1.02 | 4.54e^-2^ | 1.34 |
| 5373 | 160.02240 | 8.7 | [M+H]^+^ | 0.47 | -1.07 | 1.06e^-2^ | 1.97 |
| 5381 | 386.83075 | 1.2 | [M-H]^-^ | 2.35 | 1.23 | 5.87e^-4^ | 3.23 |
| 5444 | 252.12019 | 8.8 | [M+H]^+^ | 2.11 | 1.08 | 2.46e^-2^ | 1.61 |
| 5519 | 299.08051 | 8.7 | [M-H]^-^ | 0.41 | -1.30 | 1.60e^-3^ | 2.79 |
| 5582 | 97.06448 | 15.6 | [M-H]^-^ | 0.48 | -1.05 | 2.16e^-2^ | 1.67 |
| 5713 | 255.82204 | 8.5 | [M-H]^-^ | 0.44 | -1.18 | 1.35e^-2^ | 1.87 |

ID: identification; RT, retention time; FC, fold change

**Supplementary Table 4**. Characteristics of donors of human intestinal fibroblasts

| **Patient ID** | **Gender** | **Age (yrs)** | **Time between initial CD diagnosis and resection (months)** |
| --- | --- | --- | --- |
| 11 | Female | 58 | 14 |
| 13 | Female | 50 | 18 |
| 14 | Male | 31 | 10 |
| 15 | Male | 40 | 16 |
| 16 | Female | 70 | 14 |
| 18 | Male | 66 | 30 |
| 19 | Female | 47 | 1 |

HIF, human intestinal fibroblast; ID, identification; CD, Crohn’s disease

**Supplementary Table 5**. Primer sequences for real-time PCR

| **Gene Symbol** | **Forward Primers (5'-3')** | **Reverse Primers (5'-3')** | **PCR-efficiency** |
| --- | --- | --- | --- |
| *SDHA* | CTTGAATGAGGCTGACTGTG | ATCACATAAGCTGGTCCTGT | 92% |
| *HMBS* | AAGGGCTTTTCTGAGGCACC | AGTTGCCCATCTTTCATCACTG | 101% |
| *HK2* | CCCTGCCACCAGACTAA | GGATCAGAGCCACAACG | 89.1% |
| *SLC16A1* | CACTTAAAATGCCACCAGCA | AGAGAAGCCGATGGAAATGA | 85.2% |

*SDHA*, human succinate dehydrogenase complex A subunit; *HMBS*, human hydroxymethylbilane synthase; *HK2*, human hexokinase 2; *SLC16A1*, solute carrier family 16 member

**Supplementary Figure 1**. PCA plot the serum metabolomics of individuals and QC samples, colored according to (1) stenosis (green), (0) non-stenosis (blue) and QC samples (green). Excellent clustering of subsequent QC samples confirmed robust instrumental precision.


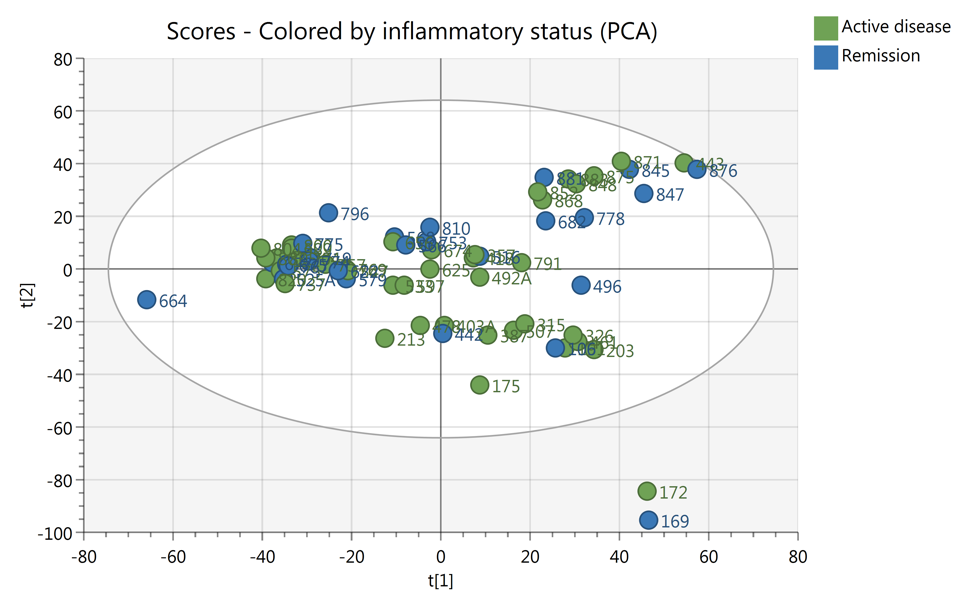

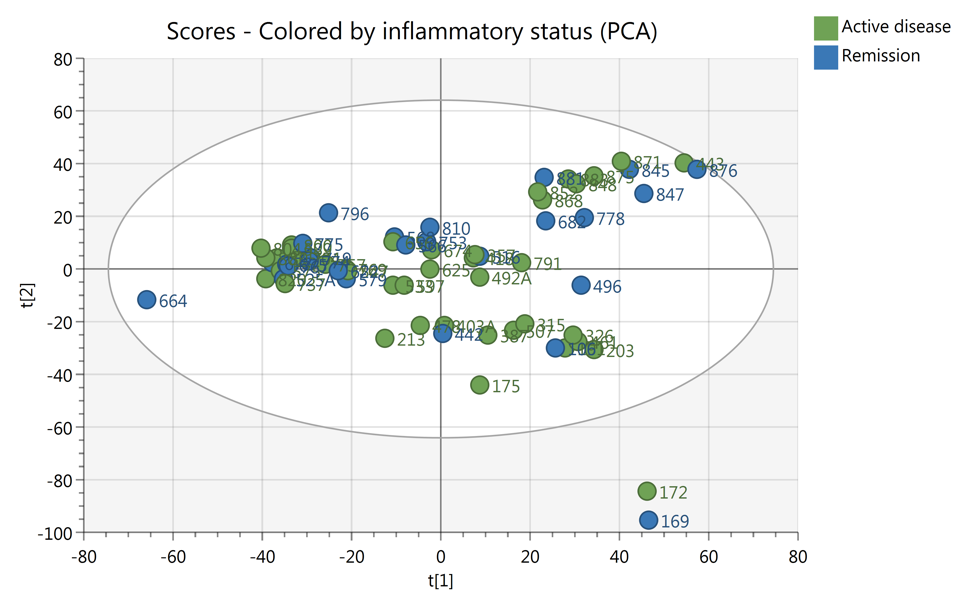

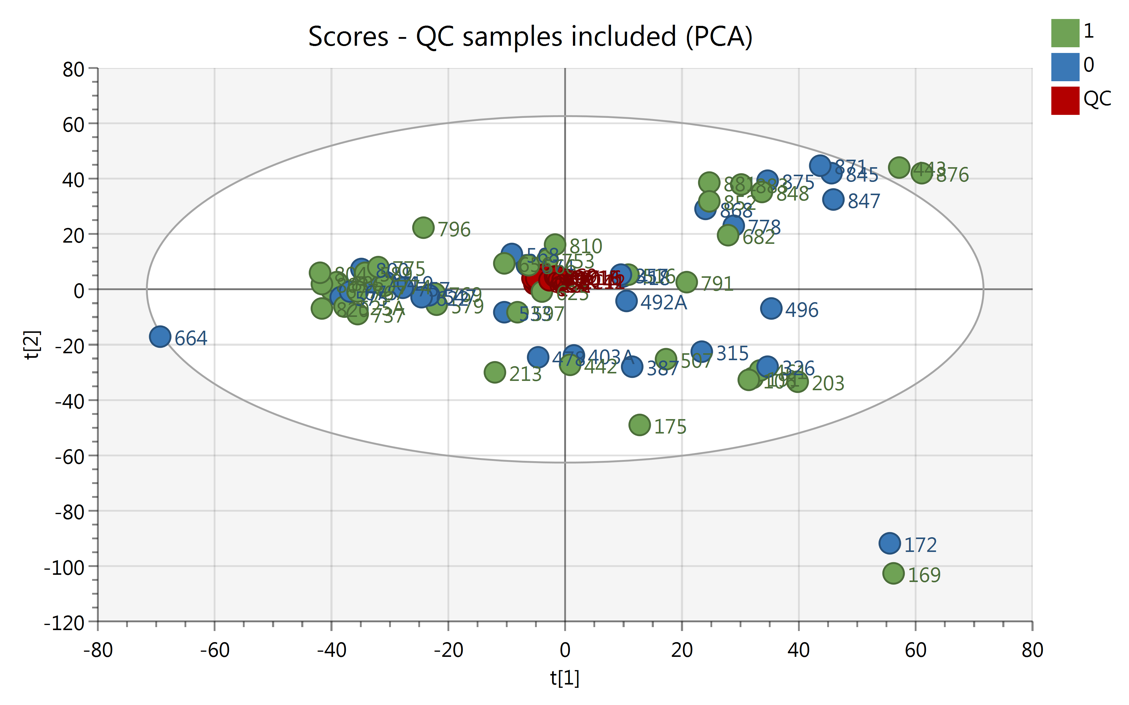

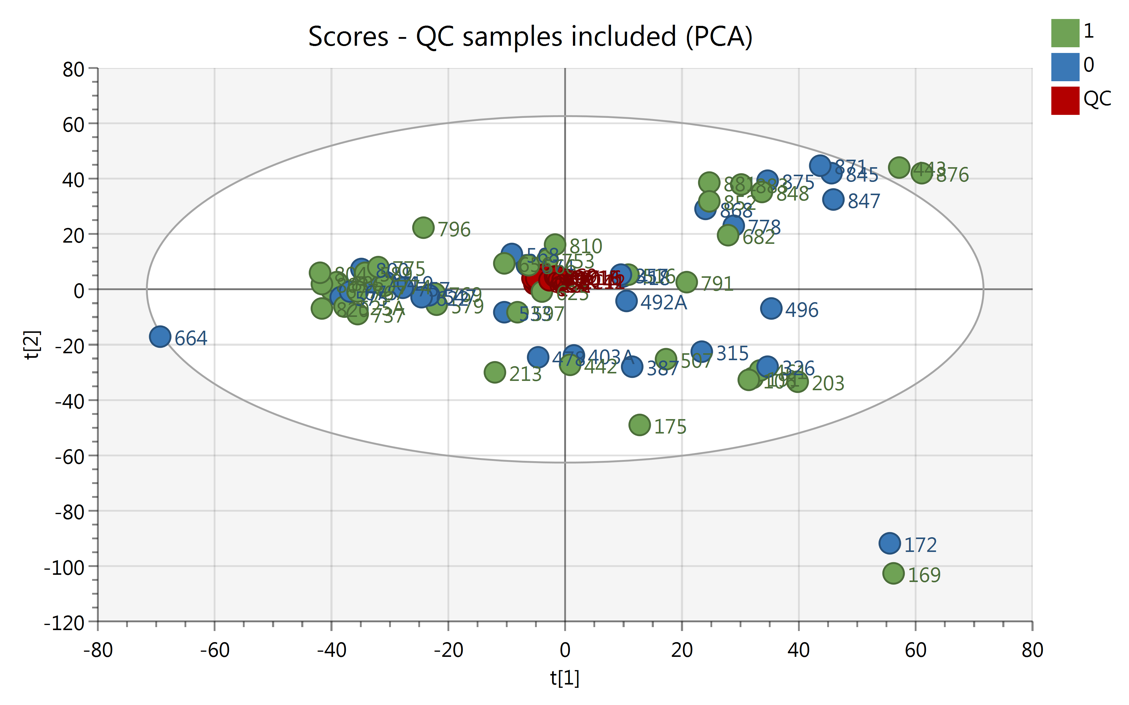


**Supplementary Figure 2**. PCA plot of untargeted metabolite fingerprints of individual patients, colored according to symptomatology (active disease (n=40) *vs.* remission (n=26)).


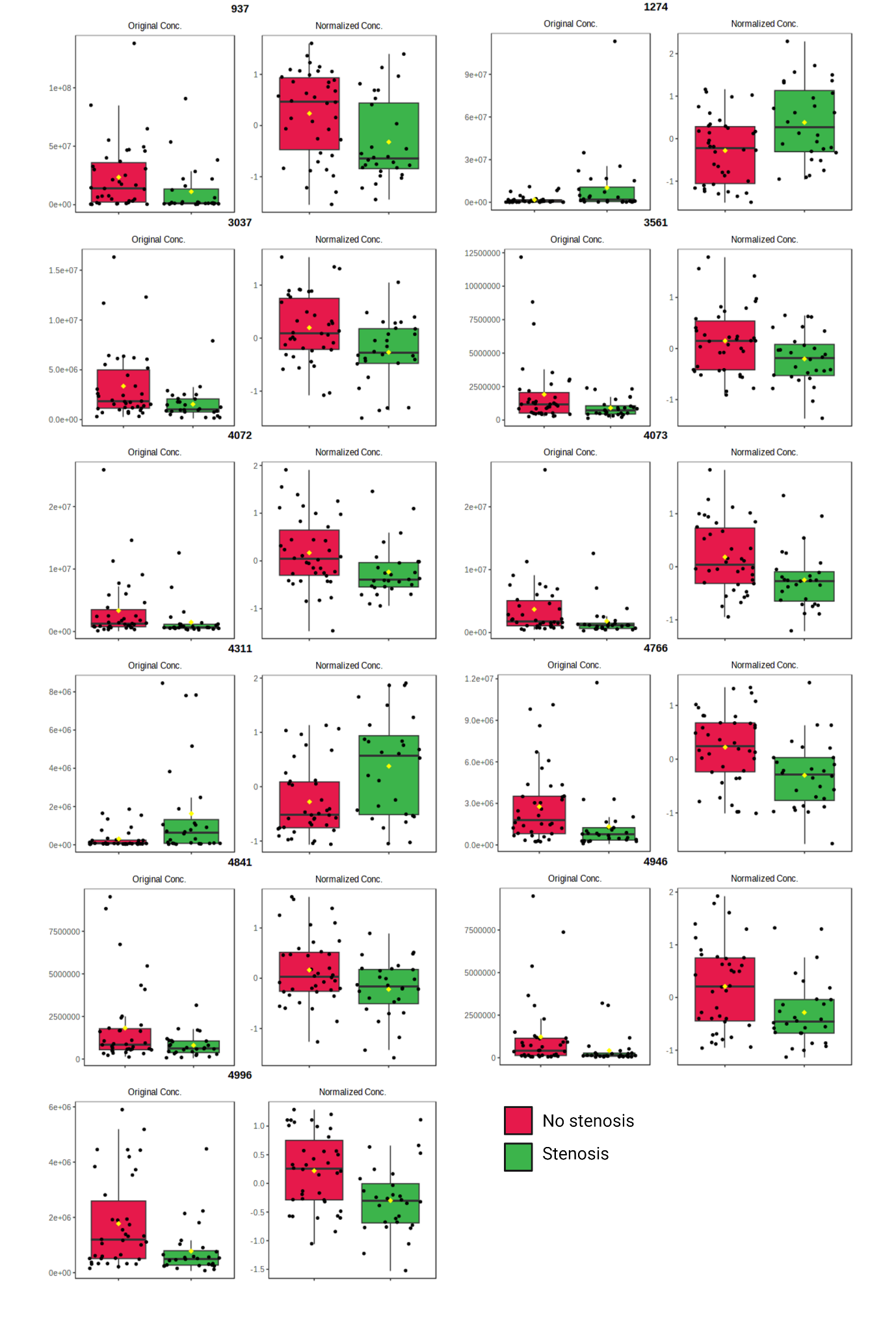


**Supplementary Figure 3.** Box plots with original and normalized concentration (in intensity values) of the top 11 differential components derived from the OPLS-DA model (Variable Importance in Projection > 1, Jacked-knife confidence interval not across zero, S-plot covariance > |0.05|) comparing sera of stenotic (n=28) versus non-stenotic (n=38) patients with CD.

*
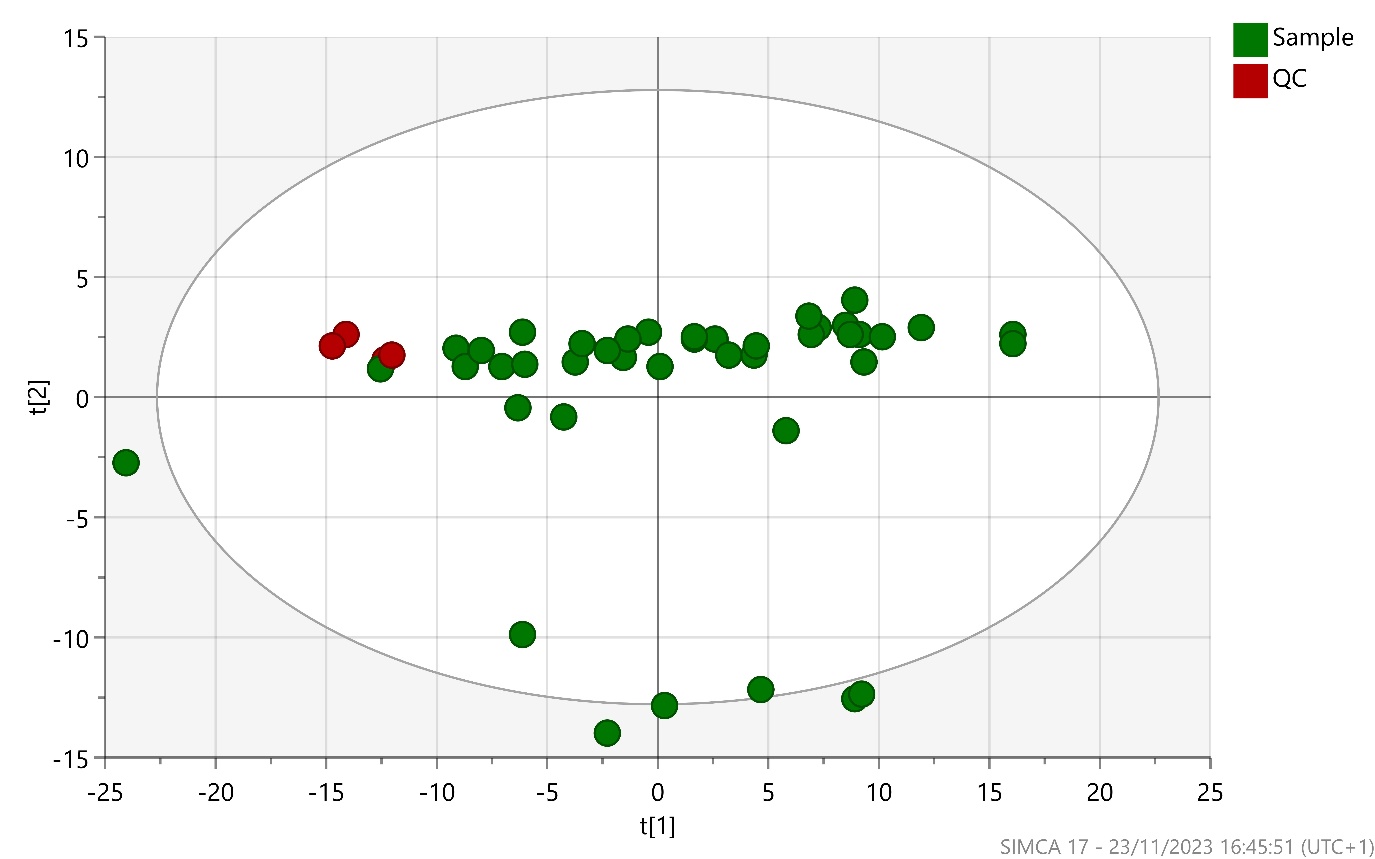
***Supplementary Figure 4**. PCA plot the cell supernatant (green) and QC (red) samples. Excellent clustering of QC samples guaranteed good instrumental precision.

**
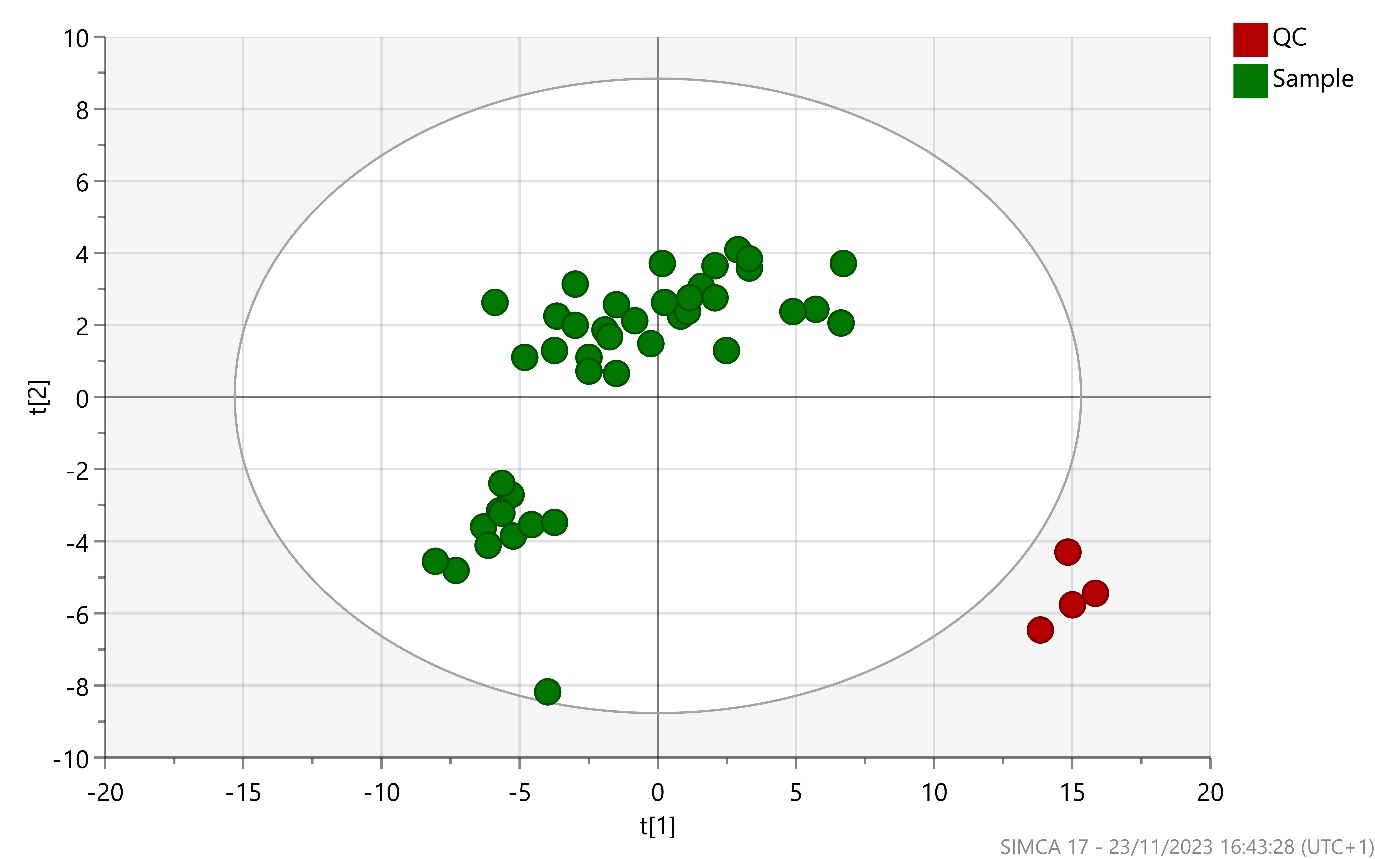
Supplementary Figure 5.** PCA plot the cell lysate (green) and QC (red) samples. Good clustering of QC samples can be observed, thereby highlighting acceptable instrumental performance.


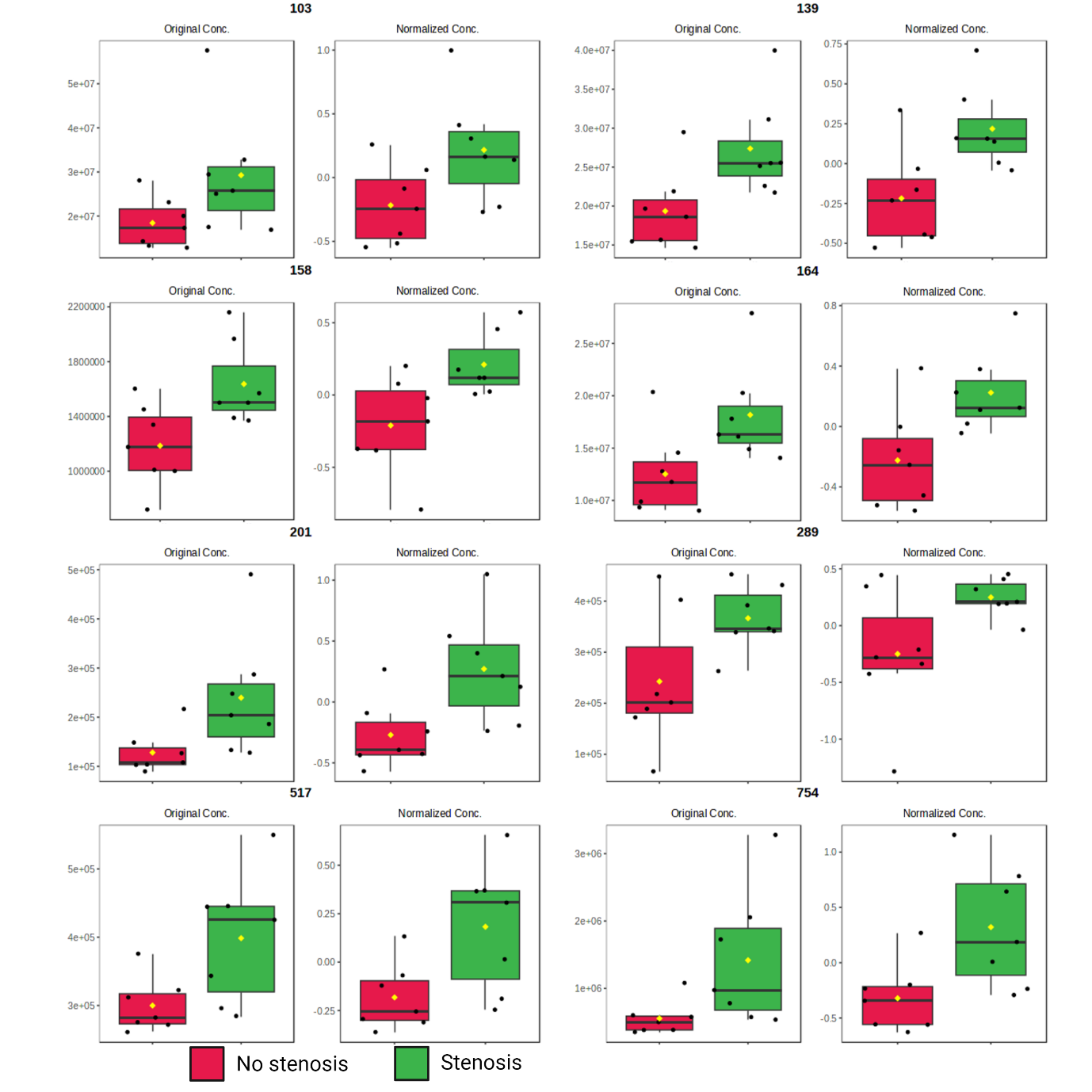


**Supplementary Figure 6.** Box plots with original and normalized concentration (in intensity values) of the differential components of cell lysates of intestinal fibroblasts of the stenotic and non-stenotic intestine.

**
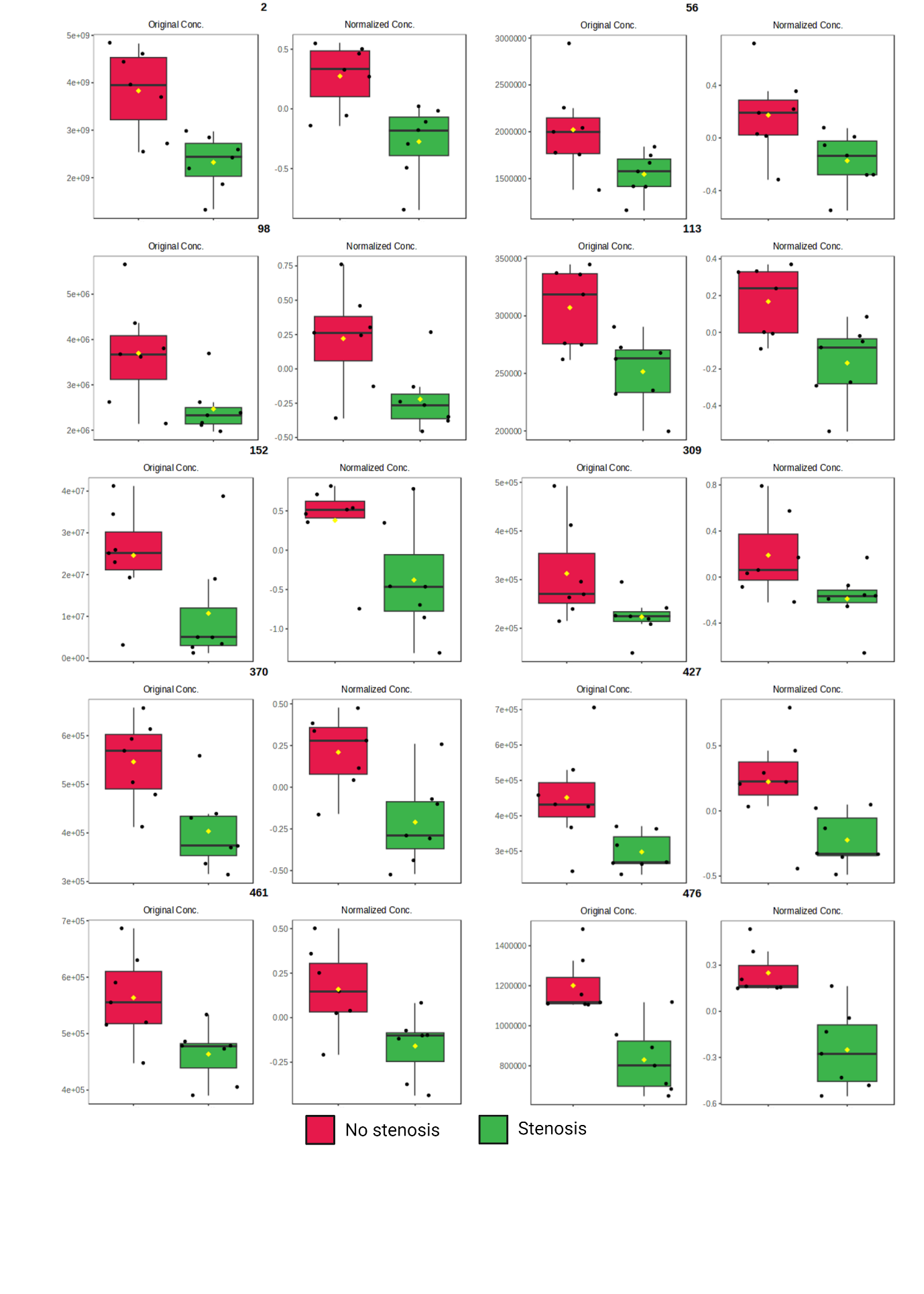
Supplementary Figure 7a.** Box plots with original and normalized concentration (in intensity values) of the differential components of the supernatant of intestinal fibroblasts of the stenotic and non-stenotic intestine.

**
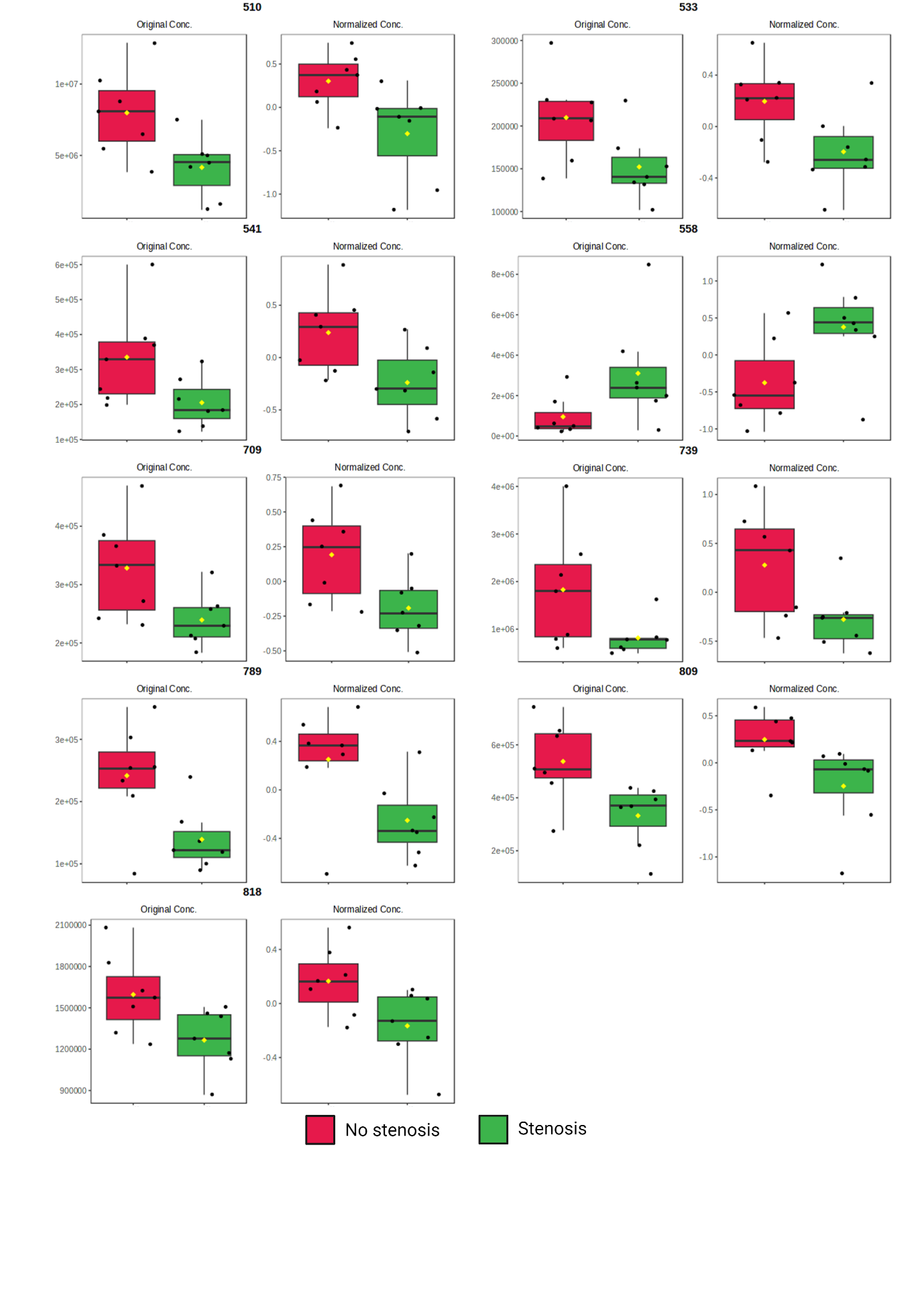
Supplementary Figure 7b.** Box plots with original and normalized concentration (in intensity values) of the differential components of the supernatant of intestinal fibroblasts of the stenotic and non-stenotic intestine.


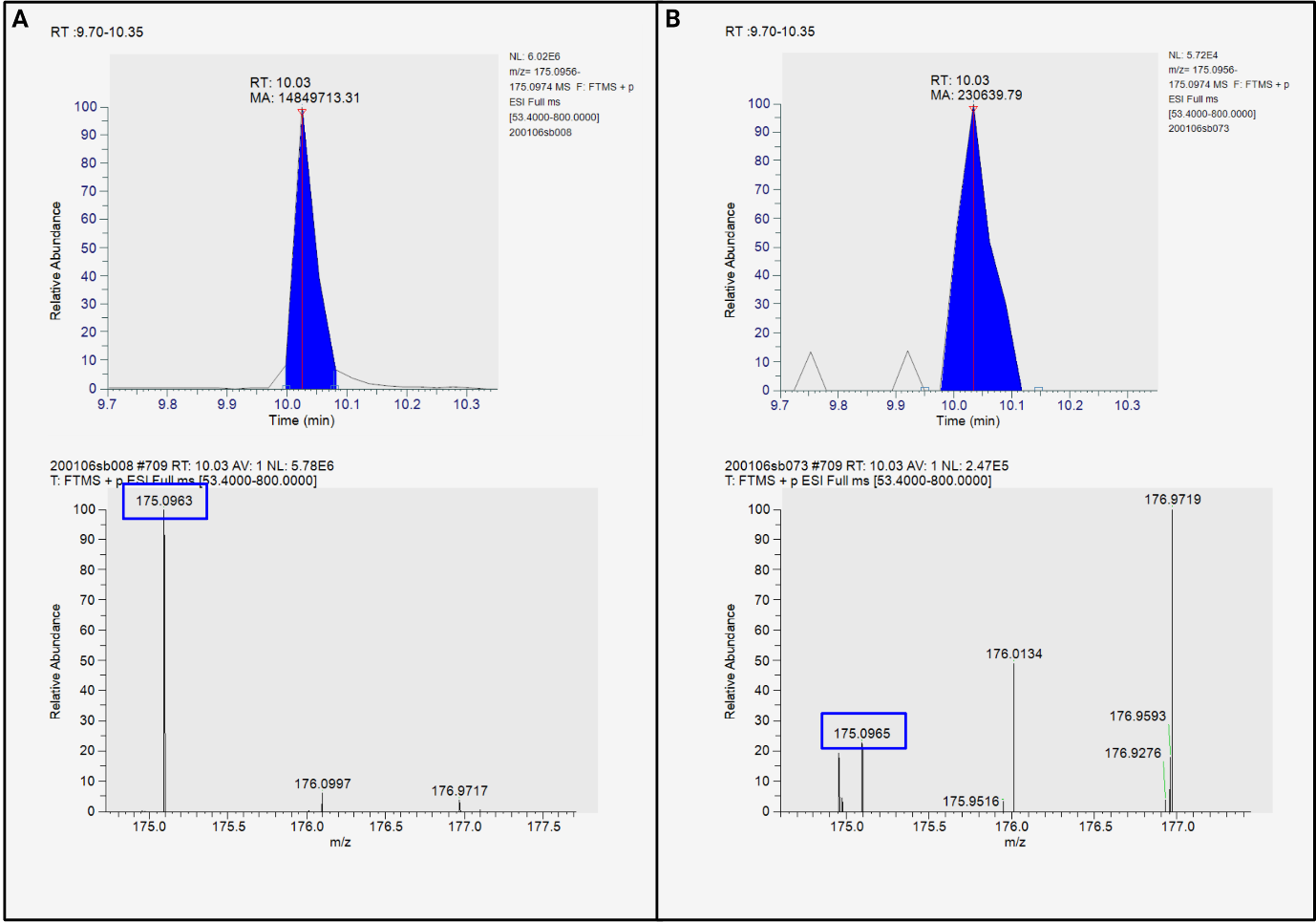


**Supplementary Figure 8.** (A) Chromatogram and MS/MS spectrum of suberic acid as analytical standard. (B) Chromatogram and MS/MS spectrum of suberic acid in a cell lysate sample.


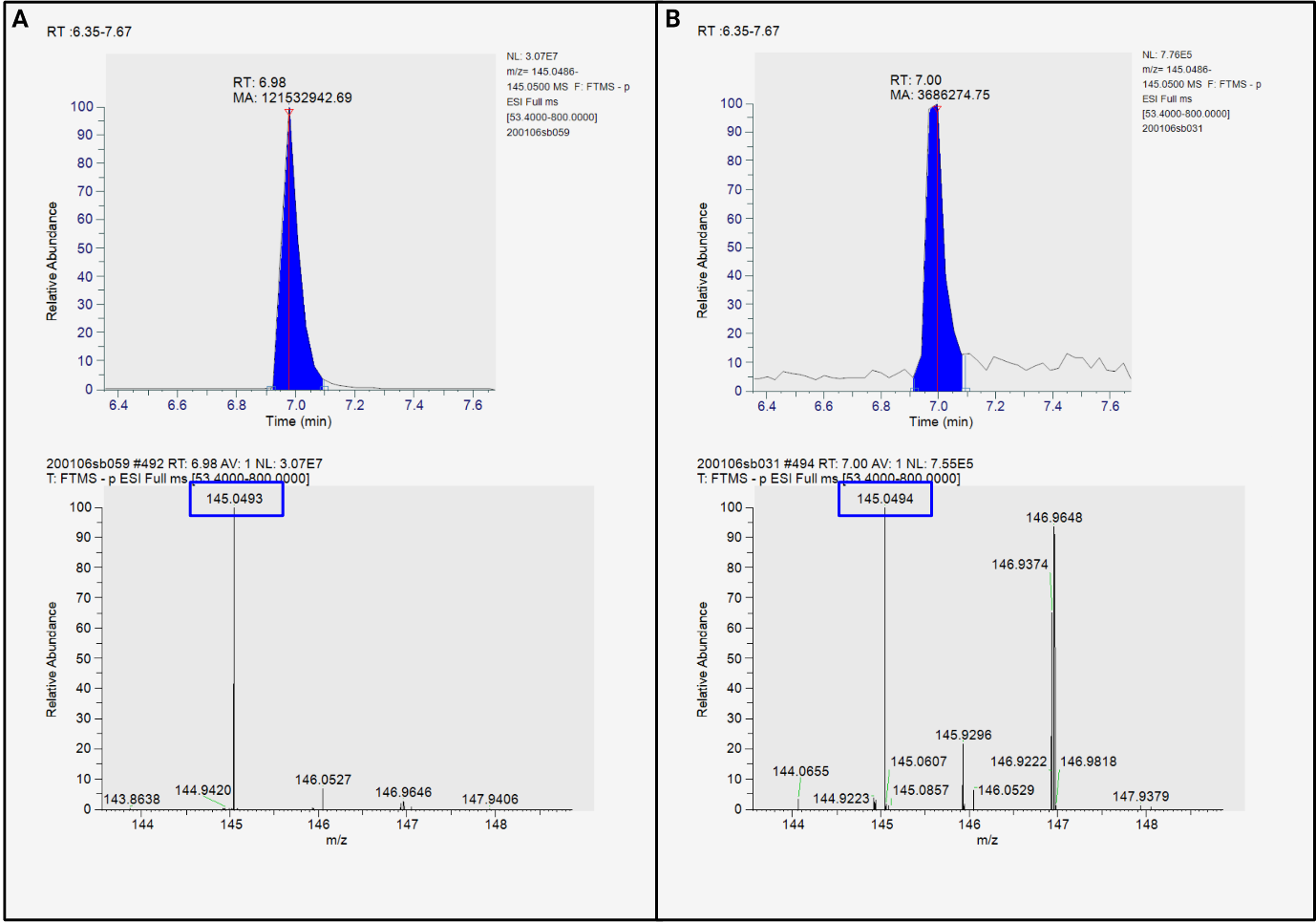


**Supplementary Figure 9.** (A) Chromatogram and MS/MS spectrum of adipic acid as analytical standard. (B) Chromatogram and MS/MS spectrum of adipic acid in a cell lysate sample.
